# Evaluating Copper-Modified Carbon Composite Nanofiber Electrodes for Electrocatalytic Nitrate Reduction

**DOI:** 10.1021/acsaenm.5c00510

**Published:** 2025-11-21

**Authors:** Ashley Hesterberg Butzlaff, Abdulsattar H. Ghanim, Yun Young Choi, Chenxu Yan, Xiaonan Shan, Nosang Vincent Myung, Charles J. Werth, David M. Cwiertny, Syed Mubeen

**Affiliations:** † Department of Civil and Environmental Engineering, 4083 University of Iowa, Iowa City, Iowa 52242, United States; ‡ Department of Chemical and Biochemical Engineering, University of Iowa, Iowa City, Iowa 52242, United States; § Department of Civil, Architectural, and Environmental Engineering, The University of Texas at Austin, Austin, Texas 78712, United States; ∥ Department of Chemical and Biomolecular Engineering, 6111 University of Notre Dame, Notre Dame, Indiana 46556, United States; ⊥ Department of Electrical & Computer Engineering, 14743 The University of Houston, Houston, Texas 77204, United States

**Keywords:** electrochemical, reduction, nitrate, catalyst support, electrode, nanofibers, resource recovery

## Abstract

The increasing urgency to address nitrate (NO_3_^–^) pollution in water sources has intensified research on electrochemical nitrate reduction, a process capable of transforming NO_3_^–^ into valuable ammonia (NH_3_) by using renewable electricity. Copper (Cu) catalysts can reduce NO_3_^–^, but their activity and selectivity toward NH_3_ can vary based on their structure, reaction environment, and support material. This study examines the efficacy of Cu-modified carbon nanofiber (CNF) supports, tailored through electrospinning, in enhancing the electrocatalytic reduction of NO_3_^–^ to NH_3_. Three variants of CNF supports were synthesized: pristine CNFs, CNFs integrated with carbon nanotubes (CNF/CNTs), and CNFs embedded with titanium dioxide nanoparticles (CNF/TiO_2_). Each electrode’s physical and electrochemical properties were analyzed before and after Cu electrodeposition. Notably, the CNF/TiO_2_/Cu composites demonstrated a selectivity exceeding 40% for the conversion of NO_3_^–^ to NH_3_ at neutral pH—significantly outperforming the CNF/CNT/Cu (<5%) and CNF/Cu (20%) configurations when deposited with equivalent amounts of Cu. The CNF/TiO_2_/Cu electrode also exhibited consistent and stable performance over the extended experimental duration (|*Q*| = 70 C), maintaining NH_3_ selectivity rates of over 50%. Tafel analysis and operando Raman spectroscopy suggest that TiO_2_ plays an active role in hydrogenating nitrogenous reduction products for enhanced selectivity. This research highlights the importance of electrode-catalyst selection in electrochemical NO_3_^–^ reduction and identifies TiO_2_-containing electrodes as promising solutions in this domain.

## Graphical abstract

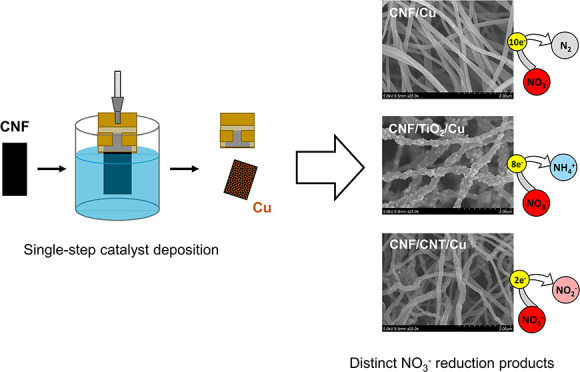

## Introduction

Electrochemical nitrate (NO_3_^–^) reduction has garnered significant research interest due to the promise of converting nitrate-rich waste streams into ammonia/ammonium (NH_3_/NH_4_^+^) and other value-added products with renewable electricity while also mitigating the environmental and health risks associated with NO_3_^–^ pollution.^1,2^ Electrochemical NO_3_^–^ reduction to NH_4_^+^ (8 electrons transferred) necessitates a highly selective and stable catalyst.^3^ Platinum group metals (PGMs) are active and stable for the NO_3_^–^ reduction reaction (NO_3_RR) but costly and in limited abundance.^4^ Transition metals provide more economic and abundant alternatives but often require complex modifications that involve additional synthesis steps and/or a PGM metal cocatalyst for improved performance.^5,6^

Although numerous studies have investigated the effect of different catalysts and catalyst modifications (e.g., doping, alloying, crystal structure, single atoms, oxygen vacancies) on the NO_3_RR mechanism and performance,^1,5^ there has been comparatively far less focus on how the nature of the catalyst support, which often also serves as the electrode, influences this process. This gap is notable given the established impact of support morphology, surface chemistry, and electronic structure on catalyst activity, selectivity, and stability.^7^ In addition, the optimal catalyst supports for electrocatalytic processes should possess high surface area for catalyst dispersion,^8,9^ high electrical conductivity for electron flow,^10^ high chemical stability (e.g., acid/base) for sustained use,^11^ and high mechanical strength for durability.^11^

Commonly used catalyst support materials for the NO_3_RR include metal oxides (TiO_2_, SiO_2_, Al_2_O_3_)^3,12,13^ and various forms of carbon (graphene, carbon nanotubes, carbon nanofibers, activated carbon),^13–15^ and work to date suggests that the nature of the catalyst support can affect the NO_3_RR, particularly in terms of product selectivity and yield. For example, titanium dioxide (TiO_2_) has shown greater NH_4_^+^ selectivity than other support materials.^16^ Moreover, TiO_2_ may provide an additional reduction mechanism due to its photoactivity,^17^ which could be exploited in photoelectrochemical systems.^18,19^ Likewise, recent work with various types of carbon supports found that certain material properties (e.g., conductivity, surface area) can influence NO_3_^–^ conversion rates.^9^ Particularly, carbon nanotubes (CNTs) hold promise to promote electrochemical performance through improved electrocatalytic activity, conductivity, and (electro)­chemical stability.^20^

In this work, we investigate carbon nanofiber (CNF) electrodes fabricated via electrospinning, a technique that allows precise control over support properties (e.g., composition, electrical resistance, surface area, porosity, catalyst distribution) as a catalyst support for electrochemical NO_3_^–^ reduction. We evaluated three different CNF-based supports each integrated with copper (Cu) nanoparticles as a catalyst: pure CNFs produced from the carbonization of polyacrylonitrile nanofibers and composites of these same CNFs embedded with either TiO_2_ nanoparticles (CNF/TiO_2_) or multiwalled carbon nanotubes (CNF/CNTs). Cu nanoparticles were integrated via electrodeposition, which can provide a defined and controlled deposition process.^21,22^ Cu was our catalyst of choice because it has shown promising performance for electrocatalytic NO_3_RR.^23–25^ For CNF composite design, we selected TiO_2_ based on observations of its superior NO_3_^–^ adsorption when oxygen vacancies are introduced^26,27^ and CNTs because of their excellent electron transport properties that were expected to lower the electrodes electrical resistance.^28–30^

Using these support formulations, we integrated Cu nanoparticles via electrodeposition and explored the NO_3_RR mechanism, rate, and product selectivity, both with and without Cu, in conditions relevant to contaminated environments or preconcentrated systems. Tafel analysis systematically evaluated the mechanism and rate-determining steps of the NO_3_RR as a function of the electrode composition. Each Cu-deposited electrode showed unique NO_3_RR performance and product distributions, where the CNF support favored NO_2_^–^ and CNF/CNT suffered from HER. Notably, CNF/TiO_2_/Cu provided the highest activity for NO_3_RR (35% NO_3_^–^ transformed after 5 C) and selectivity for ammonia (FE 42%). Moreover, in situ Raman analysis revealed that CNF/TiO_2_/Cu provided specific binding energies with NO_3_^–^ to support ammonia formation (N–H bending, out-of-plane N–H wagging). This work provides a deeper understanding of how the electrode material influences electrochemical NO_3_^–^ reduction and new insights to help guide electrode-catalyst selection for the development of high-performance electrochemical treatment and resource recovery systems.

## Materials and Methods

### Reagents

All reagents are listed in the SI.

### Electrode Synthesis

Briefly, CNF electrodes were produced via the carbonization of electrospun polymer fibers made from a precursor sol–gel solution of polyacrylonitrile (PAN) in *N*,*N*-dimethylformamide (DMF). To the above solution, phthalic acid (PTA) was included to improve solution stability and to provide fiber porosity (i.e., PTA is a known porogen when integrated into PAN-derived CNFs).^31,32^ All sol–gels were prepared with a PAN concentration of 8 wt % relative to total sol–gel mass.

Our approach to producing CNFs and CNF composites closely followed our previous work.^33^ Precursor solution composition was prepared according to eqs S1–S3. To produce CNF composites, TiO_2_ nanoparticles (Evonik P25; approximately 75% anatase/25% rutile) or unfunctionalized multiwalled carbon nanotubes (CNTs; CheapTubes) were added to the sol–gel containing DMF and PTA, prior to the addition of PAN. Based on the optimal results from our previous work,^33^ sol–gels to produce CNF/TiO_2_ composites contained 0.45 g PAN and 0.45 g TiO_2_. Sol gels to produce CNF/CNT composites contained 0.90 g PAN and 0.27 g CNT, which was the highest CNT loading found suitable for electrospinning (i.e., sol–gels did not electrospin consistently at higher CNT loadings).^34^ The specific surface area reported by the manufacturers is 50 m^2^ g^–1^ for the raw TiO_2_ nanopowder and 500 m^2^ g^–1^ for the CNTs.

Electrospun PAN nanofibers were converted to CNFs using a two-step thermal treatment process that first involved oxidative stabilization in air at 250 °C for 2.5 h, followed by carbonization in nitrogen (N_2_) at 1000 °C for 1 h. We note that the relative mass of TiO_2_ in CNF/TiO_2_ and CNTs in CNF/CNT increases after heat treatment because this sequential process is known to remove the mass of noncarbon elements.^35–37^ This thermal treatment, optimized in our previous work with CNF/TiO_2_ composites,^33^ resulted in three distinct catalyst supports, CNF, CNF/TiO_2_, and CNF/CNT, that were used as electrodes for nitrate and nitrite reduction.

### Copper Deposition on CNF Electrodes

Copper (Cu) was electrochemically deposited on substrates using a three-electrode system at room temperature, where the CNF or CNF composite served as the working electrode (WE), Ag/AgCl was the reference electrode (RE), and a platinum wire was used as the counter electrode (CE) (Figure S1). CNF-based working electrodes were secured between two glass slides with insulating tape, where one glass slide was e-beam-deposited with titanium (10 nm) and gold (80 nm) to form a conductive surface for connection to the external circuit. All deposition reactions occurred on a CNF-based electrode cut to 1.0 × 2.0 cm^2^, where 1.0 × 1.5 cm^2^ was directly exposed to the deposition solution. This resulted in a total geometric area of 3.0 cm^2^ exposed for deposition, which includes the material front and back.

The Cu deposition solution (0.3 M CuSO_4_ + 0.1 M H_3_BO_3_, pH 2.0) contained a high Cu concentration to minimize mass and charge-transfer limitations given the high surface area of the CNF-based electrodes (relative to traditional planar substrates). High hydrogen ion (H^+^) concentration has been shown to encourage Cu nucleation,^15^ accordingly, H_3_BO_3_ and pH 2.0 were used to increase H^+^ concentration. The CNF-based electrodes were submerged in approximately 25 mL of the deposition solution contained in an open beaker for the deposition. Unlike many carbon felts and foams, the CNFs did not require any pretreatment to improve wettability prior to electrochemical deposition due to their intrinsic hydrophilicity.

Copper deposition on CNF-based electrodes was conducted at a constant potential (0.12 V vs RHE), where the optimal deposition potential was first determined by linear sweep voltammetry (LSV; 2 mV s^–1^) (Figure S2). The deposition reaction was allowed to proceed until a certain amount of charge (|*Q*| = 0.229 and 0.458 C) had passed. The deposition solution provided copper in excess such that the solution-phase concentration did not appreciably change over the course of the deposition reaction (constant driving force). Following the deposition, samples were cut from the sample holder to the final dimensions (1.0 × 1.5 cm^2^) for electrodes used in nitrate and nitrite reduction experiments. Finally, deposited samples were rinsed with and placed in DI overnight to remove any residual Cu from the high-concentration deposition solution that was remaining within the CNF matrix.

### CNF and Cu Characterization

Electrochemical characterization of each CNF-based electrode was conducted prior to Cu deposition using potentiostatic electrochemical impedance spectroscopy (PEIS) (Biologic VSP-300 Potentiostat). PEIS was used to determine the uncompensated resistance for these electrodes (*R*_ohm_ = *R*_tot_ – *R*_ct_), and the results were modeled by the Randle circuit to provide an accurate fit without unnecessary circuit elements (Figure S3). PEIS also served as an additional quality control measure to confirm consistent material properties for the in-house synthesized electrodes. All resistance values (Ω-cm^2^) are reported with respect to the geometric area of the sample (0.5 cm^2^) exposed to the electrolyte (0.5 M phosphate buffer, pH 7.4), as measured in the Teflon microreactor. PEIS was conducted at open circuit voltage with the CNF or CNF composite as the WE, Ag/AgCl as the RE, and a platinum wire as the CE.

Scanning electron microscopy (SEM; S-4800 FE-SEM, Hitachi) was used to evaluate CNF and CNF composite morphology, including the average nanofiber diameter and the bulk thickness of each electrode material. Fiber diameters for each CNF-based material were measured using ImageJ (NIH), and the average and standard deviation of each diameter distribution were determined from a histogram of individual diameter measurements (n = 85). Bulk thickness was obtained via cross-sectional imaging of each electrode material, where samples were mounted on a 45/90 angled SEM stub (Edge Scientific). For each prepared cross-sectional sample, multiple measurements were recorded along the sample length, and thickness was reported as an average value (with standard deviation) of these measurements (*n* = 6). The specific surface area and pore size distribution for each CNF were measured by a surface area and pore size analyzer (Quantachrome Nova 4200e) under nitrogen. Methods to quantify surface area and pore distribution follow those outlined in our previous work.^33^

To determine the electrodeposited Cu mass (wt %), electrode samples (1.0 × 1.5 cm^2^) were dissolved in 50% (v/v) nitric acid (HNO_3_; 4 mL) following Cu deposition, and the solution was analyzed via inductively coupled plasma mass spectrometry (ICP-MS; Thermo X-series II ICPMS) (Figure S4). Because residual volumes of the concentrated deposition solution may remain within or on the surface of the nonwoven CNFs after deposition (thereby overestimating the deposited Cu mass), samples were thoroughly rinsed and placed in DI overnight to remove any residual solution. After removal of any residual deposition solution, samples were processed for ICP-MS with a sequential soak and digest method to identify the Cu resulting from electrochemical deposition alone. Samples were digested for 18–20 h (overnight), after which an aliquot of the digested solution (0.5 mL) was passed through a Nylon syringe filter (0.22 μM, 13 mm diameter; Advangene), and then diluted with DI to a final concentration of 3% v/v HNO_3_ for ICP-MS analysis.

Scanning transmission electron microscopy [(S)­TEM] with elemental mapping was used to image the distribution and morphology of Cu deposits on each nanofiber material. Samples for (S)­TEM were prepared by using a razor blade to cut off a small portion of the Cu-deposited nanofiber material. This small portion was then dispersed in ∼2 mL of methanol and sonicated for 10 min. A droplet of this methanol dispersion was then deposited onto a 300 mesh Cu TEM grid coated with a thin carbon film, and the droplet was allowed to air-dry prior to imaging. All HR-TEM analysis was carried out at the University of Notre Dame Integrated Imaging Facility using a 300 (S)­TEM CetaTM.

### Nitrate (NO_3_RR) and Nitrite (NO_2_RR) Experiments

The three CNF-based electrodes (CNF, CNF/TiO_2_, and CNF/CNT), both with and without Cu, were used for the NO_3_RR at neutral pH (pH 7) in a supporting electrolyte of 0.1 M Na_2_SO_4_. These conditions provide conservative NO_3_RR rates as SO_4_^2–^ may compete with NO_3_^–^ for adsorption sites.^38,39^ The nitrite reduction reaction (NO_2_RR) was also investigated in select electrode systems. The supporting electrolyte contained 500 ppm of NO_3_^–^ or NO_2_^–^, a concentration that would provide insight into the NO_3_RR catalyst^40^ while maintaining environmental relevance for water treatment processes, such as ion exchange (IX) brines,^41^ and concentrated sources.^42^

All reduction reaction experiments were carried out in a three-electrode, gastight divided cell (BE H-Cell 50 mL; redox.me) (Figure S5) with an ion exchange membrane to maintain pH. The CNF-based electrode (with and without Cu) served as the WE, a Saturated Calomel Electrode (SCE) was used as the RE, and a platinum wire was used as the CE. The WE was connected to the external circuit with a round tantalum clip, which was shaped in lab from a raw tantalum wire (0.02′ diameter, McMaster-Carr). Prior to transformation experiments, staircase cyclic voltammetry (SCV) was performed with all CNF-based materials (with and without Cu) in a beaker to determine the current generation range.

To prepare the cell for the reduction reaction, the cell was sealed with the lid, which contained ports for liquid/gas sampling and for all three electrodes, and purged with helium, which was also the carrier gas for gas chromatography (GC) analysis, for at least 15 min. The cell had sufficient headspace (3.75 mL) for periodic gas sampling. Reduction reactions were performed with a constant cathodic potential (−0.69 V vs RHE) applied to the WE for the duration of the experiment (determined by the total passed charge, |*Q*| = 30 C). This amount of charge was selected because it provided sufficient charge to generate a complete product distribution for gaseous and ionic products and to observe how this distribution changed with passed charge for each CNF support. For CNF/TiO_2_ electrodes, the NO_3_RR transformation was also performed at various potentials (−0.39, −0.69, −0.79 V vs RHE) and with two different Cu loadings (|*Q*| = 0.229, 0.458 C).

During the reaction, aqueous samples (0.1 mL per sample) were extracted from the WE and CE compartments as a function of passed charge (|*Q*| = 5, 10, 20, 30 C) to determine the ionic product distribution (NO_3_^–^, NO_2_^–^, NH_4_^+^). Gas samples (100 μL) were also collected as a function of passed charge to quantify gaseous product formation (H_2_) for CNF/Cu and CNF/TiO_2_/Cu. NO_3_RR product selectivity (eq S4) and Faradaic efficiency (FE) (eq S5) were determined from the mass and charge balances. pH was measured in both cell compartments after passing 30 and 70 C at −0.69 V vs RHE to determine if pH influenced reaction efficiency and/or product distribution. Lastly, in situ operando Raman spectrometry was used to identify adsorbates and reaction intermediates at the surface of Cu-deposited CNF supports under different conditions. Capturing intermediates operando as a function of applied potential could inform the NO_3_RR mechanism and hence the product distribution ratio. Additional details are provided in the SI.

The Raman instrument was equipped with a spectrometer (iHR 550; Horiba) and an inverted microscope (IX 83; Olympus). The Cu-deposited CNF supports were housed in a custom-built electrochemical reaction chamber and immersed in 0.5 M NaNO_3_. The reaction chamber is designed to control the flow rate and incident laser wavelength and intensity onto the structures for Raman signal acquisition.

### Analytical Methods

Concentrations of NO_3_^–^, NO_2_^–^, and ammonium (NH_4_^+^) were measured via ion chromatography (IC 6000; Thermo Fisher) equipped with a Dionex AS19 column for anions and a Dionex CS16 column for cations (Thermo Fisher). Gaseous H_2_ was quantified via GC with a Helium Ionization Detector (HID) and Thermal Conductivity Detector (TCD) (GC; SRI Instruments 8610C) equipped with a metal-packed column (60/80 Carboxen-1000 support, 2.1 mm ID; Supelco). Additional details of the analytical methods and equipment are provided in the SI.

## Results and Discussion

### CNF Support Characteristics

#### Bulk Structure Characterization

Figure 1 displays cross-sectional and top-view SEM images of CNF, CNF/TiO_2_, and CNF/CNT supports after carbonization at 1000 °C. Also shown are the corresponding histograms of nanofiber diameter for each material based on analysis of individual fibers (*n* = 85). The SEM analysis revealed that the average fiber diameter varied by CNF support. Average fiber diameter increased ∼1.5-fold from CNF (150 ± 30 nm) to CNF/TiO_2_ (230 ± 90 nm), consistent with behavior we have previously observed.^33^ In contrast, average fiber diameters for CNF and CNF/CNT were similar at 150 ± 30 and 140 ± 50 nm, respectively. Unlike CNF and CNF/CNT, CNF/TiO_2_ exhibited a markedly different fiber surface and relatively wide deviation in fiber diameters due to TiO_2_ aggregates within the bulk and at the surface of the fibers, which is also consistent with our prior observations.

**Figure 1:**
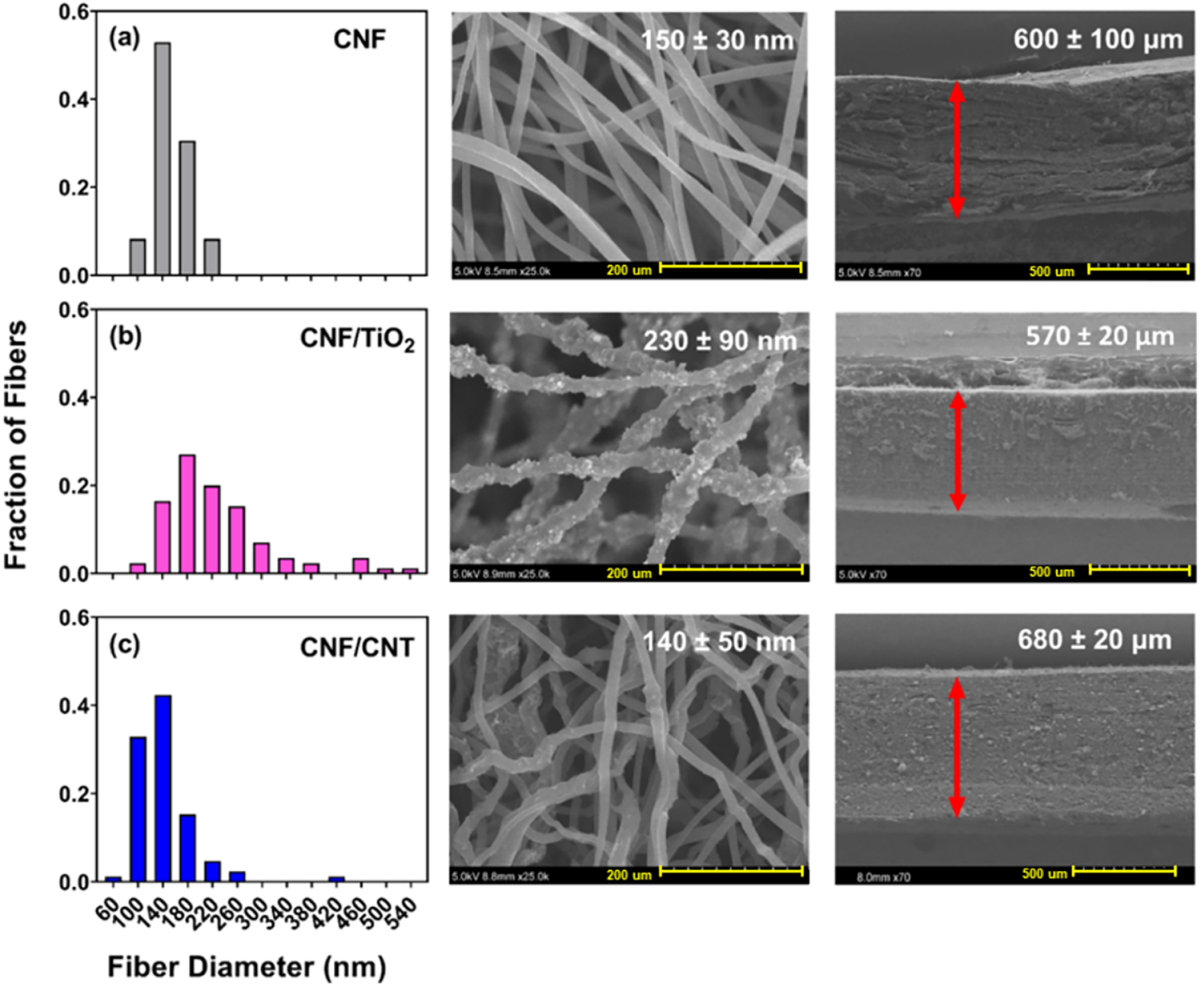
SEM images of CNF supports carbonized at 1000 °C: (a) CNF, (b) CNF/TiO_2_, and (c) CNF/CNT. Values in high-magnification SEM images represent the mean fiber diameter and standard deviation (*n* = 85). Values in cross-sectional images represent the mean thickness and standard deviation (*n* = 6). Distance between arrows identifies the electrode thickness as measured.

Table S1 shows the specific surface area and pore volume (PV) results determined from N_2_ Brunauer–Emmett–Teller (BET) adsorption isotherms (Figure S6) with each CNF-based electrode. Surface area and pore volume analysis are summarized herein, but a detailed discussion is provided in the SI. All CNF supports demonstrate type IV isotherm character and exhibit hysteresis as is typical for mesoporous materials (pore diameters from 2 to 50 nm).^43^ Overall, isotherms from the CNF supports most similarly reflect H3 hysteresis, which can be associated with irregular pores and a wide pore size distribution.^43,44^ CNF/TiO_2_ exhibited the most dramatic H3 hysteresis, which corresponds to the most delayed capillary evaporation that can be attributed to pore connectivity (i.e., larger pores surrounded by pores of smaller size or connecting necks).^44,45^ CNF/CNT isotherm character is similar to H1 hysteresis, with sharp step size at high P/P_0_ and narrow hysteresis, which can indicate better pore accessibility.^45^

CNF/TiO_2_ had the largest surface area (*S*_BET_; 54 ± 1 m^2^ g^–1^) and CNF/CNT followed closely (47 ± 3 m^2^ g^–1^), which corresponds to a roughly 5-fold and 4-fold increase, respectively, relative to CNF (11 ± 1 m^2^ g^–1^). The higher surface area and total PV for CNF/TiO_2_ and CNF/CNT likely result from the inclusion of secondary components with high specific surface area (i.e., TiO_2_, CNTs). For CNF/TiO_2_, the increased surface area and mesoporosity – which can improve mass transport (e.g., diffusion), charge transport, energy density (e.g., reaction depth), and durability^46^—were attributed to the interconnected pores and voids resulting from TiO_2_ aggregates. Although each material was synthesized and subsequently modified under identical conditions, the morphological differences and resulting electroactive area may impact the catalyst morphology (see Figure 2).

**Figure 2:**
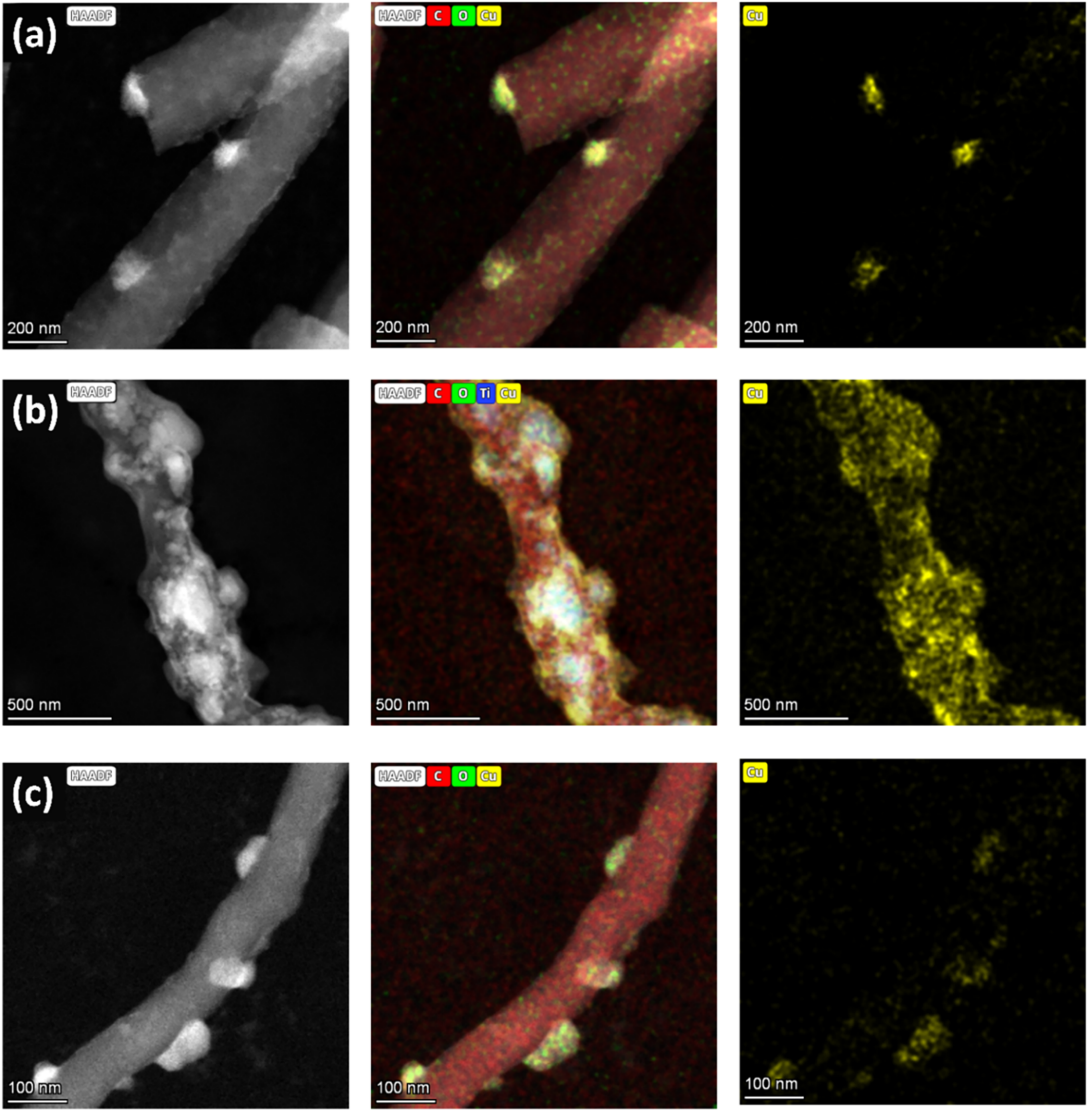
(S)­TEM images and elemental mapping of CNF supports carbonized at 1000 °C after Cu deposition at 0.12 V vs RHE, 0.458 C: (a) CNF/Cu, (b) CNF/TiO_2_/Cu, and (c) CNF/CNT/Cu.

#### Electrochemical Areal Resistance

Electrochemical characterization from PEIS measurements is presented in Table 1. The PEIS results modeled with a Randles circuit yielded similar ohmic resistances across the three supports (*R*_ohm_ ≈ 6–7 Ω·cm^2^), confirming that bulk and electrolyte contributions are comparable among electrodes. In contrast, the charge-transfer resistance showed modest but clear differences: CNF/CNT exhibited the lowest *R*_ct_ (7 ± 2 Ω·cm^2^), CNF was similar (8 ± 4 Ω·cm^2^), and CNF/TiO_2_ was higher (12 ± 2 Ω·cm^2^). The corresponding total resistances (*R*_tot_ = *R*_ct_ + *R*_ohm_) followed the same order. Thus, the support-dependent behavior arises primarily from interfacial kinetics (R_ct_) rather than bulk solution resistance (*R*_ohm_). We note that these PEIS measurements were intentionally performed before Cu deposition to isolate the electronic/interfacial properties of the supports; performance differences after Cu deposition are captured in the SCV and product selectivity analyses.

**Table 1:** PEIS Values from the Three CNF Electrodes as Modeled by the Randles Circuit

	geometric resistance (Ω cm^2^)		
CNF support	*R*_ct_	*R*_tot_	*R*_ohm_
CNF	8 (±4)	14 (±4)	6 (±1)
CNF/TiO_2_	12 (±2)	19 (±1)	7 (±11)
CNF/CNT	7 (±2)	11 (±2)	6 (±1)

We expected the CNF/CNT to have the lowest areal resistance because fibers with CNTs can achieve high specific conductivities, some similar to metal wires.^28–30^ However, it is likely that the intrinsically low resistance of pure CNTs was not fully maintained in CNF/CNT due to imperfect junctions, alignment, and spacing between CNTs within the CNF fiber.^28,47^ Although all three CNF supports exhibit slightly higher areal resistance (∼10 Ω-cm^2^) than other carbon-based electrodes (∼1.0 Ω-cm^2^),^48–51^ it should be noted that these electrodes with lower reported resistance were tested in different electrochemical systems, often at high electrolyte molarity and acid/base pH.

### Characterization of Cu Deposition for Different CNF Supports

#### Current–Time Profiles

Figure S7 displays representative current–time (*J*_geo_ versus *t*) profiles for the three CNF supports during electrochemical Cu deposition. All three CNF supports exhibited similar profiles during deposition, where the current was greatest (i.e., most negative) at initial potential onset, rapidly reached a minimum, and then increased to a near-constant current at later times. However, in contrast to CNF and CNF/CNT, the current profile for CNF/TiO_2_ reaches a minimum more slowly and remains more stable after initial potential onset.

Unlike traditional potentiostatic nucleation theory for diffusion-limited environments,^52^ the CNF deposition profiles did not exhibit the characteristic dip in current at initial potential onset that corresponds to formation of the electrical double layer. This deviation from the traditional theory (i.e., Scharifker-Hills models) could likely be due to occurrence of multiple deposition mechanisms: (1) adsorption to electrode surface (followed by diffusion and nucleation) and (2) direct attachment from liquid to existing deposits.^53,54^ Adsorption and surface diffusion can limit deposition kinetics (i.e., deposit density and growth), and dominate current contribution at early times, whereas bulk diffusion in the electrolyte can be limiting at later times.^53^ Similar experimental current–time profiles have been obtained with platinum electrodeposition in low concentration solutions; this behavior was explained by earlier growth of deposits by attachment or partial merging of deposits or diffusion regions.^55^

#### Cu Mass Loading and Morphology

Although the CNF-based electrodes provided different compositions (i.e., TiO_2_ and CNT composites) and fiber morphology (e.g., fiber diameters, surface areas, pore size distributions), ICP-MS analysis of acid-digested materials after Cu deposition revealed consistent mass loading of deposited Cu across all three electrodes (0.10 ± 0.02 mg Cu; see Table S2 and accompanying discussion). ICP-MS quality control checks are detailed in SI. The amount of deposited Cu determined experimentally by ICP-MS analysis was compared to the maximum theoretical Cu loading, which was estimated by the total charge passed during deposition (i.e., 0.458 C). Assuming a Cu valency of two (Cu^2+^), the theoretical maximum amount of Cu deposited on the CNF (0.1508 mg of Cu) is greater than what was measured via ICP-MS across all CNF substrates (0.10 ± 0.02 mg of Cu). Nevertheless, this suggests a relatively efficient electrodeposition process (∼70%). While Cu mass and deposition efficiency were comparable across all three electrodes, (S)­TEM images with elemental mapping indicate unique deposition morphologies (Figure 2). Although lattice fringes were not resolvable under the current imaging conditions, elemental mapping confirmed the dispersion of Cu nanoclusters across the CNF surface.

CNF/Cu (Figure 2a) and CNF/CNT/Cu (Figure 2c) exhibited the most similar deposit morphologies, where deposits ranged from approximately 50 to 100 nm and were primarily positioned along the fiber edge. As suggested by the different current–time deposition profile for CNF/TiO_2_, CNF/TiO_2_/Cu exhibited more dispersed and smaller Cu deposits, and elemental mapping suggests that the deposits were mostly clustered at the TiO_2_ agglomerates. This may suggest that TiO_2_ within the fiber provides better charge dispersion and surface charge for Cu deposition.

### NO_3_RR and NO_2_RR Current–Voltage Relationships

All three substrates were considered as suitable options for use in the NO_3_RR and were examined with SCV. The current density–voltage relationships (*J*_geo_ versus *E*_we_) from SCV provided insight into the reduction performance and reaction mechanism of each CNF support with (closed symbols) and without (open symbols) Cu in the N-free control electrolyte of SO_4_^2–^, NO_2_^–^, and NO_3_^–^ (Figure 3a–c, respectively). Because of similar electrical areal resistances observed for all CNF supports, we note that differences in the reduction performance across the CNF substrates were not attributed to electrical resistance.

**Figure 3:**
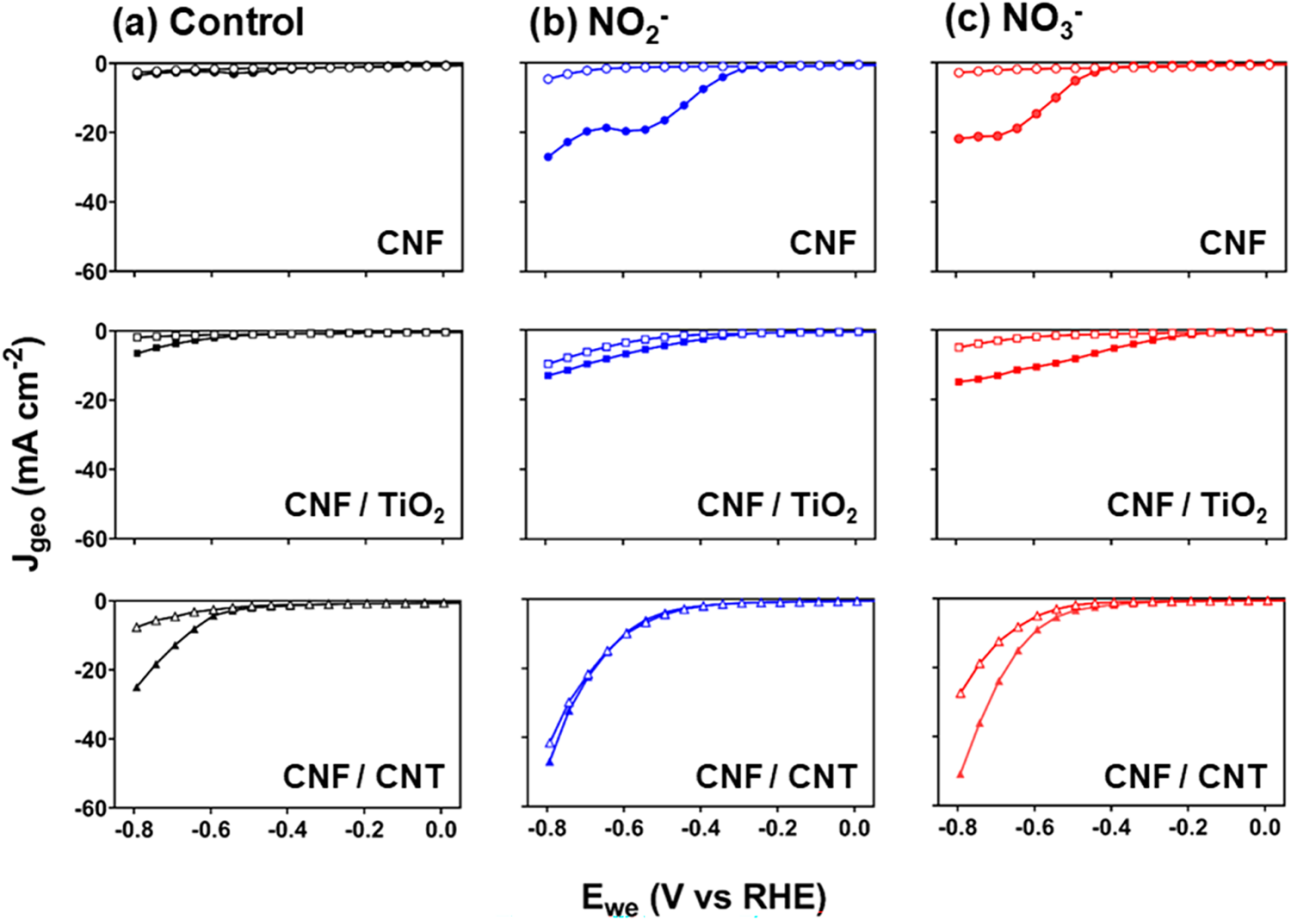
Current density–voltage profiles in (a) the N-free control electrolyte, (b) NO_2_^–^, and (c) NO_3_^–^ for the CNF supports evaluated with (■) and without (□) Cu (deposited at 0.12 V vs RHE, 0.458 C). Experimental conditions: 0.1 M SO_4_^2–^ with 500 ppm of NO_2_^–^ or NO_3_^–^, pH 7.0, purged with N_2_; 0.8 mV s^–1^ scan rate; 0.8 to −0.8 V vs RHE with IR compensation.

#### CNF Electrodes

For CNF electrodes without Cu (Figure 3, open symbols), the current density at all potentials was comparable for each electrolyte (NO_2_^–^, NO_3_^–^, and N-free SO_4_^2–^). This indicates that the cathodic currents are primarily due to HER activity and suggests that the CNF electrode is not active for NO_3_^–^ or NO_2_^–^ reduction. However, two observations were apparent with the addition of Cu to CNF: (1) a significant increase in current densities in NO_2_^–^ and NO_3_^–^ electrolyte compared to N-free electrolyte, and (2) cathodic current densities for NO_2_^–^ and NO_3_^–^ electrolyte appearing at more positive potentials compared to the N-free electrolyte. For example, the geometric current density (*J*_geo_) for CNF/Cu at −0.69 V was 8-fold greater in NO_2_^–^ and 9-fold greater in NO_3_^–^ compared to that of CNF/Cu in the N-free electrolyte and CNF without Cu in all electrolytes. The above observations unambiguously indicate that the addition of Cu to the CNF electrode provides high activity for N-species reduction. Notably, the onset potential for the CNF/Cu system occurred at a more positive potential in solutions with NO_2_^–^ compared to those with NO_3_^–^, which indicates that NO_3_^–^ reduction to NO_2_^–^ is the rate-limiting step.^12,38,56^

Further, in our reduction experiments with CNF/Cu in NO_2_^–^ and NO_3_^–^ (Figure 3b,c, closed symbols), we noted a plateau in the current at increasingly negative potentials, signaling a mass transfer limiting region. This observation, together with minimal hydrogen evolution reaction (HER) activity, suggests that the limitation at these potentials might be due to the restricted consumption or availability of electroactive hydrogen (H_ads_). The reduction process of NO_3_^–^ and NO_2_^–^ to NH_4_^+^ involves essential steps of deoxygenation and hydrogenation, both requiring H_ads_.^25,57^ Moreover, the dynamic balance between H_ads_ production and its utilization is crucial for determining the NH_4_^+^ selectivity and yield.^40,58,59^ Therefore, the plateau in the current could be indicative of a bottleneck in H_ads_ availability, which in turn affects the overall efficiency and yield of the NO_3_^–^ and NO_2_^–^ reduction to NH_4_^+^.

#### CNF/TiO_2_ Electrodes

In contrast to CNF electrodes, CNF/TiO_2_ without Cu generated currents in the presence of NO_2_^–^ and NO_3_^–^ (Figure 3b,c, open symbols), demonstrating that TiO_2_ introduces activity for NO_2_^–^ and NO_3_^–^ reduction. The reduction activity contributed by TiO_2_ is further supported by the minimal currents observed in the N-free system for CNF/TiO_2_ across all potentials (*J*_geo_ = −1.4 mA cm^–2^ at −0.69 V). CNF/TiO_2_ without Cu showed enhanced kinetics (i.e., current densities for a given potential) for NO_2_^–^ compared to NO_3_^–^, again indicating that reduction from NO_3_^–^ to NO_2_^–^ is the rate-limiting step.

With the addition of Cu in NO_2_^–^ and NO_3_^–^ (Figure 3b,c, closed symbols), reduction was initiated at more positive (less cathodic) potentials, indicating increased activity for NO_2_^–^ and NO_3_^–^ reduction. When the current exceeded −1.2 mA cm^–2^, this behavior was more evident for NO_3_^–^ (−0.19 vs −0.39 V RHE for CNF/TiO_2_/Cu and CNF/TiO_2_, respectively) than NO_2_^–^ (−0.24 vs −0.34 V vs RHE for CNF/TiO_2_/Cu and CNF/TiO_2_, respectively). Notably, the onset potential for NO_3_^–^ reduction for CNF/TiO_2_/Cu was less cathodic than that for the CNF/Cu electrode; this supports a scenario where the addition of TiO_2_ lowers the activation barrier for NO_3_^–^ reduction. The lower activation barrier for CNF/TiO_2_/Cu can be attributed to (1) increased availability of H_ads_ over CNF/TiO_2_/Cu for NO_3_^–^ reduction, as indicated by the increased HER activity observed in the N-free electrolyte (black solid squares); (2) increased stability of Cu^+^ species, which can aid in reducing the NO_3_RR overpotential; and/or (3) improved ability of TiO_2_ to directly bind, reduce, and activate NO_3_^–^ at more positive potentials.^23,26,60^

#### CNF/CNT Electrodes

CNF/CNT electrodes exhibited a higher electrochemical activity in the N-free electrolyte with and without Cu (Figure 3a). This indicates that the CNF/CNT electrodes were more active for the HER compared with CNF and CNF/TiO_2_ electrodes. While more active for the HER, CNF/CNT/Cu required a higher onset potential for both NO_2_^–^ and NO_3_^–^ reduction compared to the CNF/Cu and CNF/TiO_2_/Cu electrodes (Figure 3b,c, closed symbols). Higher onset potential demonstrates that CNF/CNT/Cu had a higher activation overpotential for NO_2_^–^ and NO_3_^–^ reduction. With increasing negative potentials, CNF/CNT and CNF/CNT/Cu demonstrated higher activity in the NO_2_^–^ and NO_3_^–^ electrolyte. However, the CNF/CNT/Cu product distribution results, presented in the subsequent sections, showed very limited reduction products. Limited product formation supports that the higher currents with a more negative potential are attributed to the HER and not necessarily NO_3_^–^ reduction.

### NO_3_RR Product Selectivity

#### Product Selectivity across CNF Supports

Figure 4 provides the distribution of ionic (NO_2_^–^, NH_4_^+^) and gaseous (N_2_, H_2_) products from NO_3_RR for CNF/Cu and CNF/TiO_2_/Cu electrodes at −0.69 V vs RHE. Product formation (μmol) is shown as the average and standard deviation of duplicate analyses (*n* = 2) per electrode type. In Figure 4, we also provide the average amount of NO_3_^–^ transformation [as a %; moles of NO_3_^–^ consumed normalized to moles of e^–^ passed (see eq S6)] on the secondary *y*-axis, whereas the average FE (%) for product formation at 30 C is provided within the corresponding bar. Table S3 presents the average NO_3_^–^ transformed (%), and Table S4 presents the average FE for the NO_3_^–^ reduction products. Because the current profiles for CNF/CNT and CNF/CNT/Cu suggested an electrode with dominant HER (resulting in poor N reduction performance), only ionic species (NO_2_^–^, NO_3_^–^, and NH_4_^+^) were quantified for this electrode type (see Figure S8).

**Figure 4:**
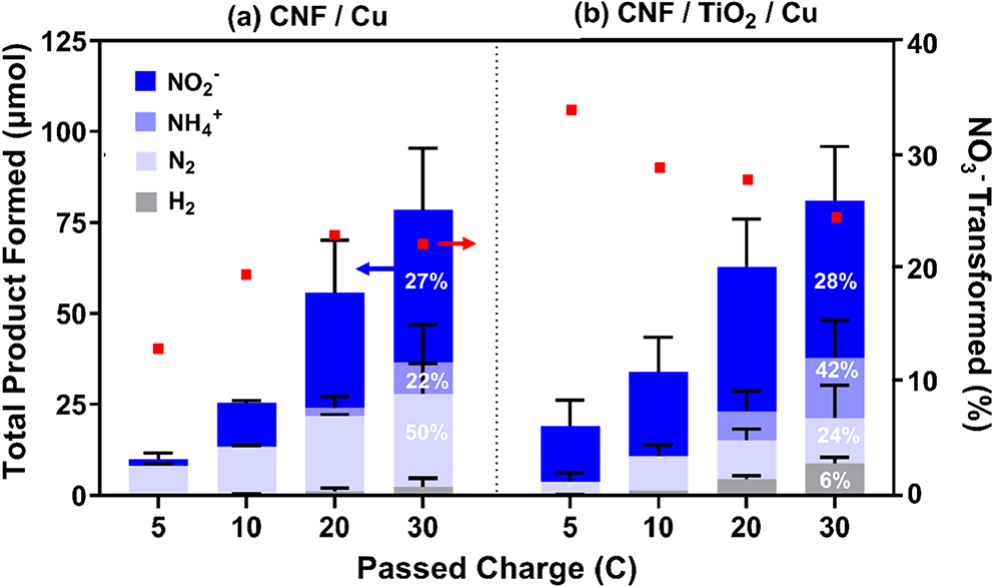
Average product distribution (primary axis) from nitrate (NO_3_^–^) reduction at −0.69 V vs RHE for (a) CNF/Cu (*n* = 2) and (b) CNF/TiO_2_/Cu (*n* = 2). NO_3_^–^ transformed (%), normalized to passed charge, is provided on the secondary *y*-axis. Faradaic efficiency (FE %) for nitrite (NO_2_^–^), ammonium (NH_4_^+^), dinitrogen (N_2_), and hydrogen (H_2_) formed at 30 C is displayed within the corresponding bar. Experimental parameters: 500 ppm of NO_3_^–^ in 0.1 M Na_2_SO_4_, pH 7.0, purged with Ar; stirred. Cu deposition at 0.12 V vs RHE, 0.458 C. Corresponding results for CNF/CNT/Cu are provided in Figure S8.

For all three CNF-based electrodes, NO_2_^–^ was the only ionic product observed when less than 20 C of total charge was passed. For total charges of 20 C or greater, NH_4_^+^ also formed, primarily in CNF/Cu and CNF/TiO_2_/Cu systems. After a total charge of 30 C was passed, which was sufficient to generate all anticipated products, CNF/Cu and CNF/TiO_2_/Cu transformed nearly the same amount of NO_3_^–^ (22% and 24%, respectively). However, CNF/TiO_2_/Cu provided the greatest FE for NH_4_^+^ (FE 42%), which also resulted in the greatest NH_4_^+^ yield (as μmol h^–1^ mg_cat_^–1^) across Cu-deposited CNF electrodes (see Figure S9a). pH change was negligible (< ± 0.1) for CNF/TiO_2_/Cu after passing 70 C, demonstrating that bulk pH did not impact NO_3_^–^ reduction or the corresponding product distribution. In contrast, CNF/Cu favored N_2_ formation across all passed charge (FE 92% at 5 C), which suggests favorable nitrogen adsorption (NO*, N*) and limited hydrogen adsorption (H*).^61,62^ However, with increasing charge, N_2_ formation became less favorable (from FE 92% at 5 C to 50% at 30 C). CNF/Cu and CNF/TiO_2_/Cu had similar FE values for NO_2_^–^ (FE = 27% and 28%, respectively) but favored different reduction products (NH_4_^+^ and N_2_, respectively). As suggested by SCV profiles, HER was more prevalent with CNF/TiO_2_/Cu (FE 6%) than with CNF/Cu (FE 2%); this implies that CNF/TiO_2_/Cu has increased H_ads_ availability to provide the NH_4_^+^-dominant product distribution. For additional reference, Table S5 provides performance metrics for other carbon-based electrodes evaluated for the NO_3_RR and NH_4_^+^ selectivity.

At the initial amount of passed charge (5 C), CNF/TiO_2_/Cu demonstrated higher NO_3_^–^ conversion and NO_2_^–^ formation (34% and 60%, respectively) relative to CNF/Cu (13% and 7%, respectively). This suggests that CNF/TiO_2_/Cu provided more favorable binding energetics for NO_3_^–^ to NO_2_^–^ reduction relative to CNF/Cu. Assuming a sequential reduction pathway (NO_3_^–^ → NO_2_^–^ → NH_4_^+^), NH_4_^+^ formation from accumulated NO_2_^–^ became more kinetically favorable with increasing passed charge for CNF/TiO_2_/Cu. As a result, CNF/Cu may provide more efficient NO_3_^–^ reduction over time (at greater total charges) due to an increasing NO_3_^–^ transformation.

For CNF/CNT/Cu, relatively low product formation and FE confirmed that HER was dominant (see Figure S8), as suggested by the current profiles (see Figure 3). At 30 C, the NO_3_^–^ transformation for CNF/CNT/Cu was only 6%, and the total ionic product was 3-fold less than CNF/TiO2/Cu, with only 4% FE for NH_4_^+^. Highly ordered pyrolytic graphite (HOPG), a reasonable analog for CNTs, has demonstrated increased HER activity with a greater number of step edges and lattice defects.^63^ The apparent HER activity of CNF/CNTs may suggest that incorporating CNTs in the CNFs introduced defects not present in the CNF.

Moreover, control experiments were conducted using the supports without Cu to help improve our understanding of the influence of catalyst addition. All three CNF-based supports without Cu produced predominantly NO_2_^–^ as the product and negligible ammonia under identical conditions (−0.69 V vs RHE after 20 C), confirming that Cu deposition is required to drive the reaction beyond the one-electron NO_3_^–^ → NO_2_^–^ step. In fact, NO_2_^–^ was the dominant product for CNF/TiO_2_ without Cu (FE 85%) and no ammonia was detected. CNF provided similar behavior, with even greater selectivity for NO_2_^–^ (FE ∼ 100%). This suggests that the inherent properties of the support material may influence reactivity for NO_3_RR but that the catalyst is influential in modulating the reaction pathway.

#### Product Selectivity with CNF/TiO_2_/Cu

Figure 5 displays the distribution of ionic (NO_2_^–^, NH_4_^+^) and gaseous (H_2_, N_2_) products as a function of applied potential (−0.39, −0.69, and −0.79 V vs RHE) for CNF/TiO_2_/Cu. Figure 5 also provides the average NO_3_^–^ transformed with passed charge (moles of NO_3_^–^ consumed normalized to moles of e^–^ passed) on the secondary *y*-axis and average FE (%) for products formed at 30 C within the corresponding bar. Figure S9b compares NH_4_^+^ selectivity in terms of yield (μmol h^–1^ mg_cat_^–1^) across this applied potential range for CNF/TiO_2_/Cu. Potential dependent studies were conducted only with CNF/TiO_2_/Cu due to its favored NH_4_^+^ formation and FE relative to those of CNF/Cu and CNF/CNT/Cu. Moreover, NH_4_^+^ selectivity was independent of pH, where the pH was nearly unchanged after NO_3_^–^ reduction.

**Figure 5:**
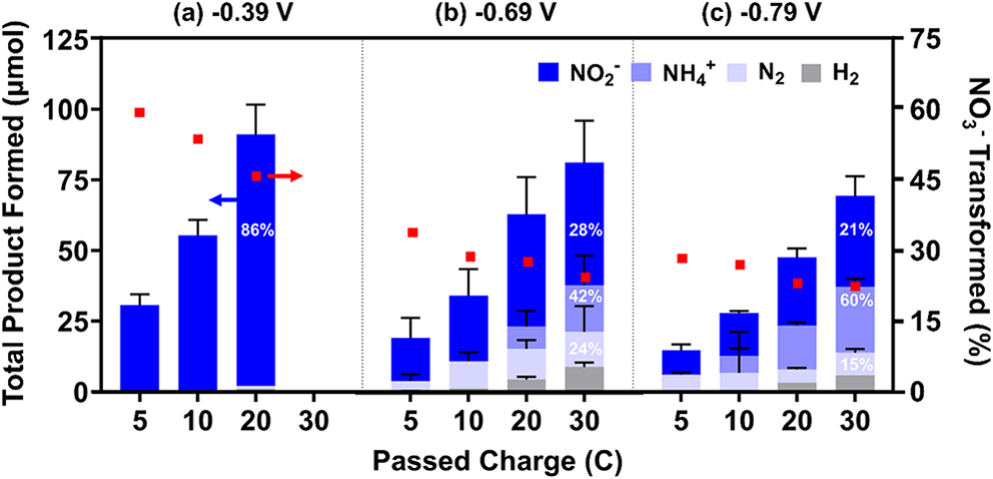
Average product distribution (primary axis) from nitrate (NO_3_^–^) reduction for CNF/TiO_2_/Cu at the applied potentials of (a) −0.39 V, (b) −0.69 V, and (c) −0.79 V vs RHE. NO_3_^–^ transformed (%), normalized to passed charge, is provided on the secondary *y*-axis. Faradaic efficiency (FE%) for nitrite (NO_2_^–^), ammonium (NH_4_^+^), dinitrogen (N_2_), and hydrogen (H_2_) formed at 30 C is displayed within the corresponding bar. FE are reported at 20 C for −0.39 V and 30 C for −0.69 and −0.79 V. Experimental parameters: 500 ppm of NO_3_^–^ in 0.1 M Na_2_SO_4_, pH 7.0, purged with Ar; stirred. Cu deposition at 0.12 V vs RHE, 0.458 C.

At −0.39 V, only 20 C were passed due to low current density, and NO_2_^–^ was the dominant product with 86 ± 10% FE at 20 C. Small amounts of N_2_ and H_2_ were generated (<15% FE combined), and no NH_4_^+^ was detected. Although −0.39 V provided the highest NO_3_^–^ transformation, dominant NO_2_^–^ formation was undesired and NO_3_^–^ transformation decreased with increasing passed charge (from 60 to 45%). The product distribution markedly changed from −0.39 to −0.69 V with a nearly 7-fold increase in current density (e.g., NH_4_^+^ FE increased from 0 to 42% from −0.39 to −0.69 V). At −0.79 V, less total product is formed due to the increased NH_4_^+^ selectivity (FE 60%). Although HER became more prominent with increasingly negative potential, NH_4_^+^ FE (60 ± 7%) was greatest at −0.79 V. The product distribution shifted from primarily NO_2_^–^ (57% FE at −0.39 V) to primarily NH_4_^+^ (60% FE at −0.79 V), which agrees well with the sequential reaction pathway (NO_3_^–^ → NO_2_^–^ → NH_4_^+^) and with trends observed elsewhere with TiO_2_/Cu for NO_3_RR.^26^ In that work, a steady increase in NH_4_^+^ and decrease in NO_2_^–^ occurred with more negative potentials.^26^

Lastly, Figure S10 provides the distribution of ionic products (NO_2_^–^, NH_4_^+^) for CNF/TiO_2_/Cu as a function of Cu deposition (|*Q*| = 0.229 and 0.458 C). We observed similar reduction currents and nearly identical ionic product distributions for 0.229 and 0.458 C, suggesting that the selected loadings do not influence the NO_3_RR kinetics or product formation for the CNF/TiO_2_ supports. Collectively, the reduction performance of the CNF/TiO_2_/Cu electrode observed herein reinforces the use of Cu as a catalyst for N-species reduction; Cu has been shown to exhibit high activity for the first rate-limiting reaction step, minimize the potential difference between NO_2_^–^ and NO_3_^–^ reduction, and limit the HER better than other metal catalysts.^1^ Moreover, Cu also performs well near the HER potential window because adsorbed hydrogen encourages NH_3_ formation over N_2_.^64^

### Long-Term Performance Trials for CNF/TiO_2_/Cu

Based on its superior performance, CNF/TiO_2_/Cu was chosen as the representative system for the detailed analysis shown in Figure 6. Less emphasis was placed on CNF/Cu and CNF/CNT/Cu at this stage, because these lacked comparable NH_3_ production efficiency and served mainly as benchmarks. When CNF/TiO_2_/Cu was subjected to a prolonged NO_3_RR period (|*Q*| = 70 C; approximately 4.8 h) at −0.69 V vs RHE (Figure 6), the electrode provided stable performance and maximized NH_4_^+^ formation in the final product distribution (50 μmol). From 5 to 20 C, the FE for NO_2_^–^ increased with passed charge. However, from 20 to 70 C, FE for NO_2_^–^ rapidly decreased and the NO_2_^–^ mass was near constant (FE ∼ 5%). This suggests that NO_2_^–^, which is identified as the first intermediate in the NO_3_RR pathway, was further reduced to the other end products (i.e., NH_4_^+^, N_2_) with increasing passed charge. In fact, NH_4_^+^ was the dominant reaction product (FE %) for the entire prolonged NO_3_RR experiment, where the maximum FE for NH_4_^+^ (∼55%) was obtained at 70 C. FE for the other primary product, N_2_, reached a maximum value at 30 C and remained constant until the experiment end (∼34%). FE for H_2_ was constant until the end of the extended period, where a 2-fold increase in FE was observed from 60 to 70 C (∼12%). The increasing H_2_ selectivity at 70 C could be attributed to the gradual depletion of nitrate and nitrite species, which leads to relatively more hydrogen evolution in later stages of electrolysis. While consistent selectivity for NH_4_^+^ was maintained throughout the extended operation, a dedicated 24 h stability test was also performed at −0.69 V vs RHE (Figure S11). The current density remained remarkably steady during this period, exhibiting less than a 10% deviation from the initial value, confirming the electrode’s long-term electrochemical stability. Nevertheless, further investigation is warranted to fully assess the practical durability and lifetime of the catalyst under continuous operation. As observed at 30 C, the bulk pH remained essentially unchanged (ΔpH < ±0.1) after passing 70 C, indicating negligible bulk electrolyte alteration over the test duration.

**Figure 6:**
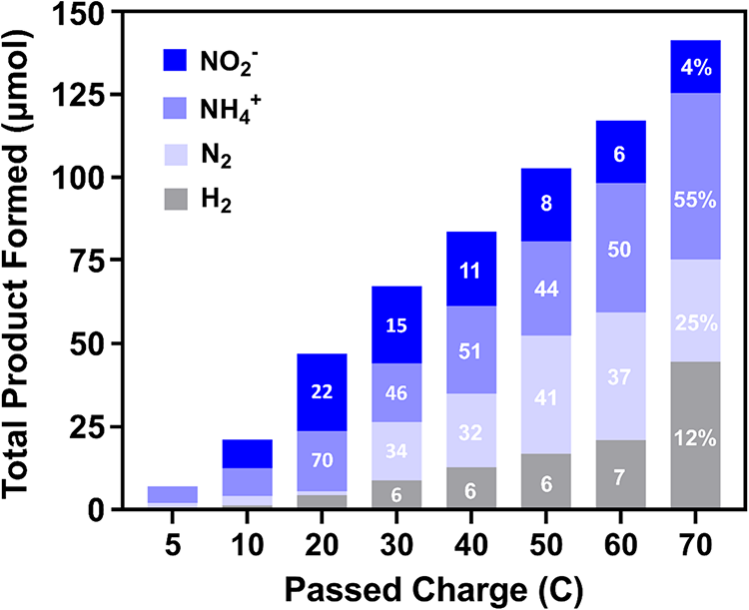
Product distribution (primary axis) from nitrate (NO_3_^–^) reduction for CNF/TiO_2_/Cu at −0.69 V vs RHE for an extended duration (|*Q*| = 70 C). Faradaic efficiency (FE %) for nitrite (NO_2_^–^), ammonium (NH_4_^+^), and dinitrogen (N_2_) formed at each charge increment is displayed within the corresponding bar. Cu deposition at 0.12 V vs RHE, 0.458 C. Experimental parameters: 500 ppm of NO_3_^–^ in 0.1 M Na_2_SO_4_, pH 7.0, purged with Ar; stirred.

### Mechanistic Insights into the NO_3_RR on Cu-Modified CNF Electrodes

#### Tafel Slope Comparison

To gain insight into the reaction mechanism for NO_3_^–^ reduction on the Cu-deposited CNF supports, we constructed Tafel plots (log |*J*_geo_| versus *E*_we_) (Figure 7a, top row).^65^ The Tafel analysis focused on more negative cathodic potentials (−0.89 to −0.19 V vs RHE), where the generation of electroactive hydrogen (H_ads_) is sufficient for the NO_3_^–^ (and NO_2_^–^) reduction reaction.^66^ Importantly, in our approach, we compared Tafel slopes (Figure 7b, bottom row) across supports to infer the corresponding reduction mechanism, rather than extracting precise kinetic parameters like exchange current density (*i*_0_), which can often lead to inaccuracies.^67,68^

**Figure 7:**
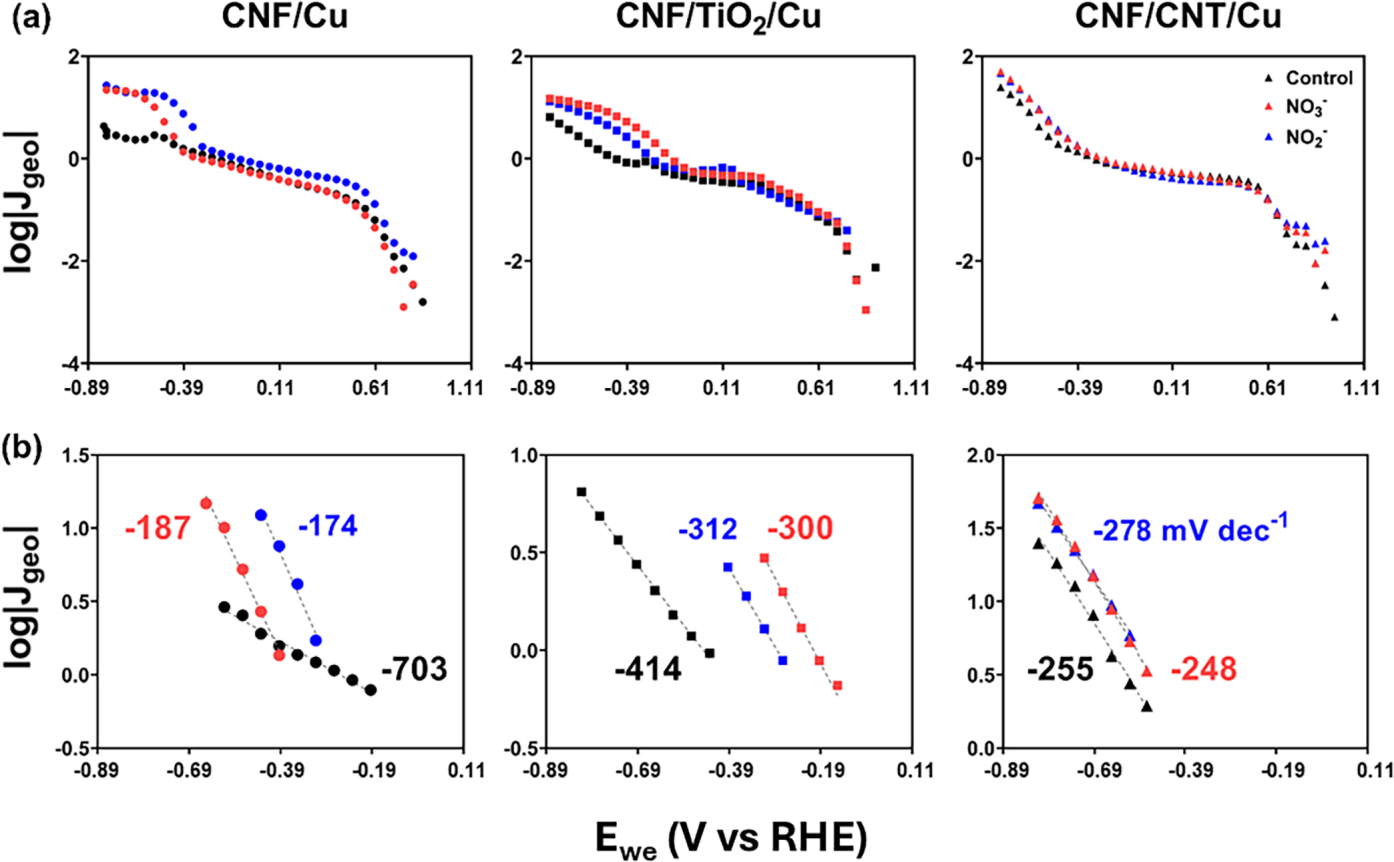
(a) Tafel plots (log|*J*_geo_| versus *E*_we_) and (b) Tafel slopes from a narrow potential range in the N-free control electrolyte (black), NO_2_^–^ (blue), and NO_3_^–^ (red) systems for the CNF/Cu (left), CNF/TiO_2_/Cu (middle), and CNF/CNT/Cu (right). The potential range for the reported Tafel slopes was determined by an onset potential where *J*_geo_ = 0.75 mA cm^–2^.

Tafel analysis reveals important differences among the CNF-based substrates that influence the NO_3_RR. CNF/TiO_2_/Cu exhibited an intriguing pattern; the Tafel slope in the N-free electrolyte (414 mV dec^–1^), which reflects the HER, was slightly larger than those observed during NO_3_^–^ and NO_2_^–^ reduction. The comparatively large Tafel slope observed in the absence of nitrate likely reflects intrinsic kinetic limitations for HER on the Cu-modified CNF electrode, conducive for the conversion of NO_3_^–^ to NH_4_^+^.^23,24^ In the presence of nitrate, H_ads_ consumption was promoted through nitrate reduction pathways, which lowered the apparent slope and reflected the balance between the availability of H_ads_ and its utilization in competing reactions.

In contrast, the CNF/Cu system showed a significantly larger Tafel slope in the N-free electrolyte (703 mV dec^–1^). This unusually large Tafel slope is more likely due to the intrinsic properties or surface kinetics of the Cu–CNF catalyst (e.g., a slow step in the HER mechanism on this surface). This agrees with the lower Faradaic efficiency for NH_4_^+^ observed in our product analysis, suggesting a more limited H_ads_ availability. As a result, N_2_ was the most favorable product for CNF/Cu because less hydrogenation steps were required for its formation. For the CNF/CNT/Cu system, the Tafel slope in the N-free electrolyte was even more favorable (255 mV dec^–1^), implying an excess supply of H_ads_. This excess likely leads to the dimerization of H_ads_ atoms, culminating in hydrogen evolution rather than efficient utilization for nitrate reduction.

### Operando Raman Spectroscopy Analysis for Reaction Pathway Determination

To further elucidate the reaction pathway, operando Raman measurements were carried out on the CNF/TiO_2_/Cu electrode (selected for its high NH_4_^+^ selectivity of ∼55%). Figure 8 presents the Raman spectra of CNF/TiO_2_/Cu during NO_3_^–^ reduction as a function of applied potential. At the most positive potential (0 V vs Ag/AgCl, ∼0.60 V vs RHE), only a single prominent band near 1050 cm^–1^ is observed, which corresponds to the symmetric NO_3_^–^ stretching mode of solution-phase nitrate. This confirms that at open circuit or mild potentials, mostly unreacted NO_3_^–^ is detected in the interfacial region.

**Figure 8:**
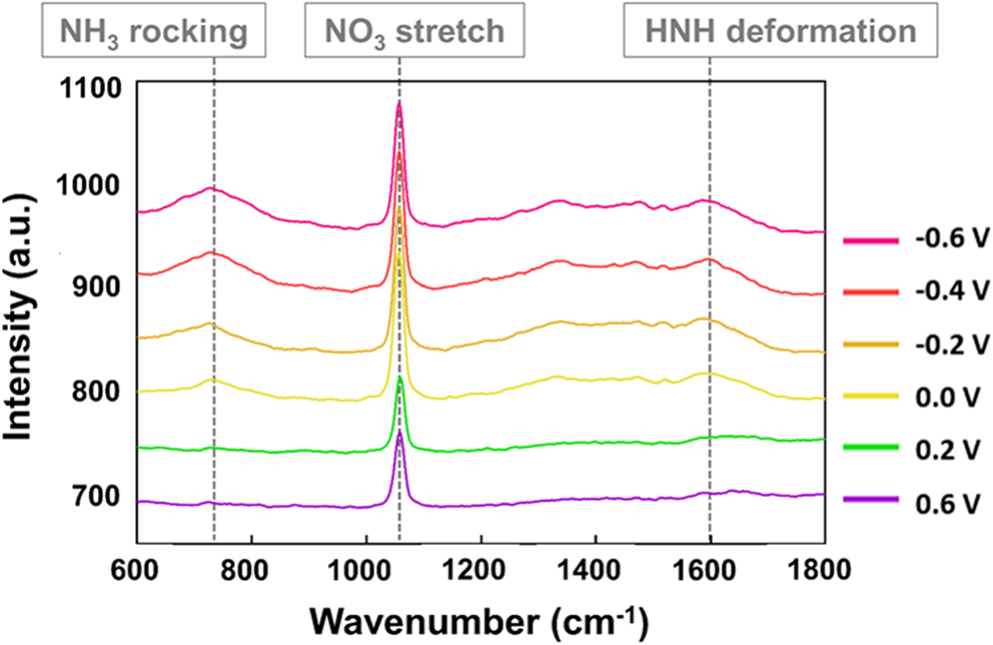
In situ operando Raman spectra as a function of applied potential (V vs RHE) in 0.5 M NaNO_3_^–^ with CNF/TiO_2_/Cu.

As the potential is scanned more negatively (increasing overpotential for NO_3_^–^ reduction), new Raman peaks emerge and grow in intensity. Notably, bands appear at approximately 1310 cm^–1^ and 710 cm^–1^, and these become more pronounced at greater cathodic potentials. Based on prior reports, the ∼1310 cm^–1^ feature can be assigned to an N–H bending (H–N–H deformation) mode of an ammonia species, while the ∼710 cm^–1^ band is attributable to the rocking vibration of NH_3_ (out-of-plane N–H wagging). The increase of these N–H vibration bands with increasingly negative potential indicates that the formation of N–H-containing products (ultimately NH_3_/NH_4_^+^) is favored at higher overpotentials.

Signals attributable to other possible intermediates such as nitrous oxide (N_2_O) or hydroxylamine (NH_2_OH) were not clearly resolved in our spectra. However, we note that prior studies have observed transient NH_2_OH-related Raman features near ∼1525 cm^–1^ under highly reducing conditions on Cu surfaces.^69^ In our spectra, although a faint shoulder may begin to appear in this region at the most cathodic potentials, its intensity remains low and its assignment remains tentative. This suggests that if NH_2_OH forms, it likely does so only transiently and is rapidly consumed en route to NH_3_. The absence of a strong or persistent NH_2_OH signature supports a mechanism favoring direct surface hydrogenation with minimal accumulation of free hydroxylamine.

Raman results therefore point toward a reaction mechanism in which adsorbed nitrogen species are directly hydrogenated to NH_3_ on the surface, consistent with a Langmuir–Hinshelwood-type hydrogenation pathway. Notably, both Langmuir–Hinshelwood and Eley–Rideal routes may involve proton-coupled electron transfer; however, in this mechanism, NO_3_^–^ (or its NO* fragment) likely remains adsorbed on the surface and is incrementally hydrogenated by surface-bound H atoms (H*), yielding NH_3_. This contrasts with a mechanism in which a solution-phase proton reacts with an adsorbed NO*_x_* intermediate to form NH_2_OH.

Previous operando studies of Cu electrodes support this interpretation. For example, adsorbed NO_3_^–^, NO_2_^–^, and NO were identified as the key intermediates en route to NH_3_ on oxide-free Cu, and that facile NO adsorption and reduction are critical for high NH_3_ yield.^70^ The spectroscopic assignments in our study (e.g., 1310 cm^–1^ band) also agree with recent findings, which identified Raman bands in the 1300–1370 cm^–1^ region as the symmetric H–N–H bending modes of NH_3_ formed on Cu catalysts.^71^

Importantly, the trends revealed by operando Raman spectroscopy correlate with both the Tafel analysis and the long-term NO_3_RR product distribution in this work. Tafel analysis (Figure 7) indicated that TiO_2_ in the CNF support modulates the availability of adsorbed hydrogen (H*), which in turn affects the NO_3_RR mechanism. In the N-free (pure HER) electrolyte, the Tafel slope for CNF/TiO_2_/Cu (∼414 mV dec^–1^) was between that of CNF/Cu (much larger, ∼703 mV dec^–1^) and CNF/CNT/Cu (smaller, ∼255 mV dec^–1^). This indicates that the TiO_2_-containing electrode can generate a moderate surface H* coverage – neither too low to hinder hydrogenation nor so high as to favor H_2_ evolution exclusively. Such balanced H* availability is ideal for a Langmuir–Hinshelwood mechanism, which requires sustained coupling of H* to adsorbed NO*_x_* species. Continued discussion, comparing the mechanisms of all three Cu-modified electrodes, is provided in the SI.

Overall, the operando Raman analysis provides direct spectroscopic confirmation that NO_3_^–^ reduction on CNF/TiO_2_/Cu proceeds predominantly via a hydrogenation-driven Langmuir–Hinshelwood-like pathway to yield ammonium. The spectral identification of adsorbed NO_3_^–^ and the emergence of N–H bending/rocking signals of NH_3_ at negative potentials support a mechanism in which NO_3_^–^ is first adsorbed and reduced to an NO or N intermediate on the surface, followed by stepwise addition of H to form NH_3_. This mechanistic picture coherently ties into the Tafel data (implicating the involvement of adsorbed hydrogen in the rate-determining steps) and the observed product trends (greater NH_4_^+^ formation when H is sufficiently available).

## Conclusions

In response to existing material challenges limiting the electrochemical reduction of nitrate (NO_3_^–^) to value-added ammonia (NH_3_), we herein demonstrate an electrode design tailored for the NO_3_RR to leverage potential catalyst/support interactions for improved performance. We fabricated three different CNF formulations using electrospinning and assessed their performance for electrochemical NO_3_^–^ reduction in a divided cell at neutral pH using Cu as a cost-effective metal catalyst. The three CNF electrodes were CNF/Cu, CNF/TiO_2_/Cu, and CNF/CNT/Cu, where TiO_2_ and CNT were added to the CNF matrix during electrospinning and Cu was electrodeposited post-electrospinning. Despite consistent catalyst mass loading and similar electrical resistance across all three supports, each Cu-deposited electrode showed unique NO_3_RR performance and product distributions. For all three electrodes, Cu addition proved critical to driving NH_3_ formation, advancing reduction beyond the single electron transfer step (NO_3_^–^ → NO_2_^–^)

CNF/TiO_2_/Cu transformed the most NO_3_^–^ (75 μmol) with the greatest NO_3_^–^ to NH_3_ selectivity (NH_4_^+^ FE 42%) after passing 30 C at −0.69 V vs RHE. Moreover, CNF/TiO_2_/Cu sustained NH_3_ selectivity (NH_4_^+^ FE 55%) with nearly unchanged bulk pH after an extended reduction period (70 C) at the same potential. This product distribution, along with the mechanistic insight provided by the Tafel slopes, suggests that TiO_2_ and TiO_2_/Cu play a unique role in the NO_3_RR kinetics and mechanism. Moreover, embedded TiO_2_ nanoparticles helped improve the catalyst morphology (i.e., smaller deposits) and dispersion (i.e., more widely distributed on embedded TiO_2_ surfaces) when integrating Cu via electrochemical deposition. In contrast, CNF/CNT/Cu showed the highest currents and the lowest FE for NH_4_^+^ after passing 30 C at −0.69 V vs RHE. While high hydrogen evolution activity served as a detriment for the NO_3_RR with CNF/CNT/Cu, hydrogen evolution promoted NH_4_^+^ formation and NO_3_^–^ conversion with CNF/TiO_2_/Cu. Accordingly, we suggest that CNF/TiO_2_/Cu has increased NH_4_^+^ selectivity with the formation of a hydrogenated nitrogenous species.

This work adds to the growing body of literature suggesting the unique performance of TiO_2_-based materials for NO_3_^–^ reduction and the important role of catalyst–support interactions in tuning electrochemical performance. It would be valuable to integrate alternative cost-effective catalysts with the CNF/TiO_2_ support and compare the resulting catalyst lifetimes and NH_4_^+^ yields. In the future, we intend to evaluate the Cu catalyst lifetime and further explore the properties of TiO_2_ that impart its favorable activity toward NO_3_^–^. We also plan to investigate the (photo)­electrochemical (PEC) activity of CNF/TiO_2_/Cu for NO_3_RR, which would leverage the photoactivity of the electrode as demonstrated in our previous work.^33^ Overall, we demonstrate the design and use of CNF supports as potential electroactive frameworks to boost the performance of the electrocatalytic reduction of NO_3_^–^ to NH_3_.

## Supplementary Material



## Notes

Additional detail and discussion: Materials and Methods, Results and Discussion; Tables S1–S5; Figures S1–S11; and equations S1–S6 (PDF)
