# Supplementary material for: Evaluating Copper-Modified Carbon Composite Nanofiber Electrodes for Electrocatalytic Nitrate Reduction
Source: ACS Appl Eng Mater. 2025 Nov 21;3(12):4282–95. doi: 10.1021/acsaenm.5c00510 (PMC12750526; doi:10.1021/acsaenm.5c00510)
Supplement: Supplementary file 1 [file em5c00510_si_001.pdf]

SUPPORTING INFORMATION

Evaluating Copper-modified Carbon Composite Nanofiber Electrodes for Electrocatalytic Nitrate Reduction

Ashley Hesterberg Butzlaff,[a] Abdulsattar H Ghanim,[b] Yun Young Choi,[d] Chenxu Yan,[c] Xiaonan Shan,[e] Nosang Vincent Myung,[d] Charles J. Werth,[c] David M. Cwiertny,[a*] Syed Mubeen[b*]

[a] Department of Civil and Environmental Engineering, University of Iowa, Iowa City, Iowa 52242, United States

[b] Department of Chemical and Biochemical Engineering, University of Iowa, Iowa City, Iowa 52242, United States

[c] Department of Civil, Architectural, and Environmental Engineering, The University of Texas at Austin, Austin, Texas 78712, United States

[d] Department of Chemical and Biomolecular Engineering, University of Notre Dame, Notre Dame, Indiana 46556, United States

[e]Dept. of Electrical & Computer Engineering, The University of Houston, Texas 77204, United States

* Corresponding authors E-mail: david-cwiertny@uiowa.edu; syed-mubeen@uiowa.edu

Prepared for *ACS Applied Engineering Materials*

5 tables, 11 figures

MATERIALS AND METHODS

Reagents

All reagents were used as received. The electrospinning precursor solutions were synthesized with polyacrylonitrile (PAN; MW 150,000, Aldrich), phthalic acid (PTA; Aldrich), and N,N-dimethylformamide (DMF; 99.85%, BDH Chemicals). To synthesize composite carbon nanofibers (CNFs), precursor solutions contained titanium (IV) oxide (TiO_2; Aeroxide P25, Evonik) or carbon nanotubes (CNTs; CheapTubes, 030401). Aeroxide P25 is a crystalline nanopowder mixture (approximately 75 wt. % anatase, 25 wt. % rutile) synthesized by vaporizing titanium tetrachloride, mixing with air and hydrogen, and reacting at high temperature in a burner.[1] Potentiostatic electrochemical impedance spectroscopy (PEIS) was carried out in 0.5 M potassium buffer (anhydrous potassium phosphate monobasic, KH_2PO_4; and dibasic, K_2HPO_4; Research Products International) prepared from deionized water and pH-adjusted to neutral pH with phosphoric acid (85 wt% aqueous solution; Aldrich) if needed. The Cu deposition solution was prepared with copper(II) sulfate pentahydrate ($CuSO_4 \cdot 5H_2O$, 98%+; Aldrich) and boric acid (H_3BO_3, ACS grade; Fisher). After complete mixing, pH was adjusted to 2.0 with 2.0 N sulfuric acid (H_2SO_4, ACS grade; Fisher). For staircase cyclic voltammetry (SCV) and nitrate/nitrite reduction reaction (NRR) experiments, sodium nitrate ($NaNO_3$, ACS Reagent; Aldrich) or sodium nitrite ($NaNO_2$, ACS grade; Fisher) was added to 0.1 M sodium sulfate (Na_2SO_4, ACS reagent; Aldrich) and pH-adjusted to

neutral pH with 0.1 M sodium hydroxide (NaOH) if needed. Similarly, $NaNO_3$, $NaNO_2$, and ammonia sulfate ($(NH_4)_2SO_4$, ACS grade; EM Science) were added to Na_2SO_4 to prepare calibration standards for ion chromatography (IC). Calibration standards for gas chromatography (GC) were performed with an analytical grade standard of mixed gases consisting of CO_2, CO, H_2, CH_4, and O_2 at 1 mol% each in N_2 (Supelco, Cat. No. 23463).

Electrode synthesis

Solution preparation and electrospinning. Electrospun CNF electrodes were derived from a sol gel containing polyacrylonitrile (PAN; 8 wt. % relative to total solution mass) as the polymer precursor and N, N-dimethylformamide (DMF) as the suspending solvent. Based on our previous work, phthalic acid (PTA) was included to produce mechanically stable and durable CNFs with increased flexibility from introduced porosity.[2]

Precursor solution composition was determined by the following:

$$m_{PTA}(g) = \frac{wt\%_{PTA} * [m_{P25}(g) + m_{PAN}(g) + V_{DMF}(mL) * \rho_{DMF}(\frac{g}{mL})]}{(100 - wt\%_{PTA})} \quad \text{(S1)}$$

$$wt\%_{P25} = \frac{m_{P25}(g)}{(m_{P25}(g) + m_{PAN}(g))} \quad \text{(S2)}$$

$$wt\%_{CNT} = \frac{m_{CNT}(g)}{(m_{CNT}(g) + m_{PAN}(g))} \quad \text{(S3)}$$

First, TiO_2 (or CNTs) were mixed with PTA + DMF and sonicated (Branson 510) for five hours prior to the dissolution of PAN. Second, PAN was added to the suspended TiO_2 (or CNTs) and thermomixed at 60 °C and 700 rpm (2 h for TiO_2; 5 h for CNTs).

Precursor solutions were stored in capped vials on an end-over-end rotator after sonication (Branson 510) and/or thermomixing (Eppendorf ThermoMixer C). If not used immediately after preparation, solutions were thermomixed again for 30 minutes at 60 °C and 700 rpm prior to electrospinning to remove any remaining solid PAN.

Next, the solution was transferred to a 12 mL plastic syringe (HSW Norm-Ject), which was connected to 2.0 mm ID polyethylene (PE) tubing via a PE 1/16" female luer lock fitting (NanoNC Co., Ltd). The fitting was secured to a metal nozzle adapter (Nano NC, Korea) while the opposite end of the nozzle adapter was connected to a 1/4" metal dispensing needle (JG23-0.25HPX, Jensen Global). The nozzle was secured in a custom-made stand. The solution was pumped through the tubing, nozzle adapter, and needle at a rate of 0.5 mL/h via a syringe pump (New Era Pump Systems, Inc.). After exiting from the needle, the solution entered an electric field, produced from a positive applied electric potential difference of 15kV (high voltage AC to DC converter, Acopian). The applied potential between the needle tip and the grounded rotating metal drum collector (SPG Co., Ltd, Korea, 9-5/16" circumference, approximately 600 rpm) causes CNF deposition by forming a whipping jet at the needle tip and evaporating the solvent. Nanofibers were deposited on aluminum foil covering the rotating drum collector. Needle tip was positioned 10 cm from the rotating metal drum collector. The total time for electrospinning (7-20 h) depended on solvent volume, as determined by the sol-gel formula.

Stabilization and carbonization. Oxidative stabilization and carbonization were carried out in a programmable tube furnace (MTI Corporation, OTF-1200X-UL) with a quartz tube (90 mm diameter). Temperature programming was achieved with the built-in

high precision digital controller (±1 °C). Although the controller is highly accurate, the heating zone exhibits a temperature distribution across the length of the tube. Samples were centered in the furnace below the thermocouple, where temperature reached its maximum and closest value to the set value. The actual temperature varied from the set temperature by approximately ±45 °C.

Nitrate (NO3RR) and nitrite (NO2RR) experiments

For NRR transformation in the gas-tight cell (**Figure S5**), aqueous samples were extracted (0.1 mL per sample) from the working electrode (WE) and counter electrode (CE) compartments during the transformation period to quantify ionic products via IC (Dionex ICS-6000 Dual Channel System; Thermo Scientific). The IC system was equipped for analysis of both anionic and cationic species with two separate columns (Dionex AS19-4µm, Dionex CS16-4µm). Nitrate and nitrite concentrations were determined using an isocratic method with the following parameters: 0.250 mL/min, 20 mM KOH, 13 mA, 30 ºC column, and 5 µL injection volume. Ammonium concentration was determined using a multi-step eluent gradient method with the following parameters: 0.160 mL/min, 55 mM MSA, 26 mA, 35 ºC column, and 10 µL injection volume.

Similarly, gaseous samples were extracted (50 or 100 uL per sample) from the cell headspace of the WE compartment during the transformation period to quantify gaseous products via GC. The GC (SRI Instruments 8610C) was operated under helium flow with an oven temperature of 75 °C. The GC was equipped with a HID set at 200 °C

and a TCD set at 100 °C. The total runtime of 7 minutes was sufficient for the detection of all products.

To identify and quantify NO3RR transformation, product selectivity (%) and Faradaic efficiency (FE, %) were calculated. Product selectivity was defined as:

$$S_x = \frac{m_x}{\Delta m_{NO3-}} \times 100\% \quad \text{(S4)}$$

where x denotes the product of interest, m_x is the product mass as measured (mol), and Δm_{NO3-} is the total change in NO_3^- mass for a given passed charge. Faradaic efficiency (FE, %) was defined as:

$$FE_x = \frac{q_x}{q_{tot}} \times 100\% = \frac{(m_x \times n \times F)}{q_{tot}} \times 100\% \quad \text{(S5)}$$

where x denotes the product of interest, q_x is the charge consumed by the species of interest (C), m_x is the mass of the product measured (mol), n is the number of electrons to reduce 1 mol NO_3^- to the desired product (mol), F is Faraday's constant (96485 C mol^{-1}), and q_{tot} is the total charge passed by the potentiostat (C). NO_3^- transformation (conversion) was normalized to the passed charge:

$$C_{NO3-} = \frac{\Delta m_{NO3-}}{m_{e-}} \times 100\% \quad \text{(S6)}$$

where m_{e-} denotes the total moles of electrons and Δm_{NO3-} is the total change in NO_3^- mass (moles) for a given passed charge.

NO3RR selectivity and Faradaic efficiency (FE) were calculated from the mass and charge balances, which were provided by the ionic products (NO_2^-, NH_4^+) quantified by IC and the gaseous product (H_2) quantified by GC (see **Equations S4** and **S5** above). We assume N_2 was the only other gaseous product formed during the NO3RR because no peaks were detected when the GC analytical method runtime was

extended. In fact, microporous carbons have been shown to exhibit strong sorption of N_2O,[3] which some have suggested provides additional time for reduction to N_2 (via the Vooys-Koper Mechanism)[4] if N_2O were to form. Moreover, N_2O formation is likely only in high NO_3^- concentrations (> 0.1 M) and/or acidic conditions.[4, 5] Accordingly, the reported N_2 mass was calculated based on a complete charge balance after quantifying all other products (NO_2^-, NH_4^+, H_2).

SUPPLEMENTAL RESULTS AND DISCUSSION

CNF support characteristics

Additional surface area and pore volume details. Surface area and pore volume analysis for CNF/TiO_2 was provided in our previous work, which followed the same synthesis and analytical procedures herein.[6] As the relative pressure (P/P_0) approaches unity, the adsorbed gas quickly increases due to capillary condensation in the mesopores.[7] Each CNF provided different step sizes with P/P_0, where the CNF/CNT isotherm rose most drastically at high P/P_0 to indicate rapid capillary condensation.

Consistent with their greater specific surface area, CNF/CNT (0.16 ± 0.006 cm^3 g^{-1}) had the greatest total PV followed by CNF/TiO_2 (0.10 ± 0.001 cm^3 g^{-1}). CNF/TiO_2 and CNF/CNT had similar pore size distributions with over 90% of the volume composed of mesopores, but CNF/CNT had a greater proportion of macropores (5 ± 0.6%) than CNF/TiO_2 (3 ± 1%). CNF/CNT also had an average mesopore diameter over 4-fold greater than that for CNF/TiO_2 (9 versus 2 nm, respectively). Accordingly, the smaller S_{BET} and greater total PV of CNF/CNT relative to CNF/TiO_2 is presumably attributed to its larger pores (i.e., a smaller surface-area-to-volume ratio) and/or less

surface roughness. Plain CNFs had the smallest total PV (0.02 ± 0.005 cm^3 g^{-1}), an order of magnitude smaller than for CNF/CNT and CNF/TiO_2, which too corresponds to its relatively low S_{BET}. Compared to CNF/TiO_2 and CNF/CNT, CNF also exhibited a pore size distribution with less volume attributed to mesopores (86 ± 5%) and more volume attributed to micropores (5 ± 1%) and macropores (9 ± 5%).

Cu deposition characterization across CNF supports

Additional details of Cu mass loading from ICP-MS. From ICP-MS analysis, dissolved Cu was measurable in all the DI soak solutions used to remove any non-deposited Cu retained with the CNFs after deposition. The greatest Cu mass recovered in the DI soak solution was obtained from CNF/TiO_2 after deposition (1.6 ± 0.5 mg or 0.3% of the total Cu in solution during deposition). This mass is equivalent to approximately 80 µL of the Cu deposition solution being retained on and/or within the fiber matrix after deposition.

Control samples, which were dipped in the deposition solution for the same amount of time as required for deposition but without applied potential, were also digested to account for any Cu on the CNF supports resulting from sorption or electroless deposition. For the control samples, approximately 0.02 mg Cu was detected by ICP-MS across all three CNF substrates. This mass indicates the Cu retained by the fibers when exposed to the deposition solution and without electrochemical deposition; this mass was used to correct (via subtraction) the Cu mass quantified on the electrochemically deposited substrates.

Operando Raman Spectroscopy Analysis

From Tafel analysis and *operando* Raman, the CNF support alone showed a deficient H* supply (reflected by its large HER Tafel slope, ~703 mV dec^{-1}), corresponding to its lower NH_4^+ Faradaic efficiency (more difficulty in hydrogenating intermediates). In contrast, the CNF/CNT support had an excess H* (low HER slope, ~255 mV dec^{-1}) which likely led to premature H_2 generation from H* recombination rather than productive hydrogenation of nitrogen species. The TiO_2-modified electrode hits a “sweet spot” (intermediate Tafel slope, ~414 mV dec^{-1}), where H* is generated and consumed in situ by NO_3^-/NO* adsorbates, maximizing NH_3 production. This interpretation is bolstered by the long-term electrolysis results: over an extended charge (70 C) the CNF/TiO_2/Cu showed increasing NH_4^+ selectivity and H_2 evolution, with decreasing NO_2^- in the product mix. Initially, NO_3RR on Cu can produce some NO_2^- (from incomplete reduction), but as the reaction proceeds, NO_2^- (and its NO* intermediate) is continually hydrogenated to NH_3 on the TiO_2/Cu surface. The consumption of NO* in forming NH_4^+ means fewer NO* are available to recombine into N_2 or form N_2O, directing the pathway toward ammonia. Once the supply of nitrogen intermediates becomes rate-limiting (at high conversion or charge passed), any surplus H* simply produces H_2 – explaining the rise in H_2 at high charge. Such behavior – a shift to H_2 when nitrate/nitrite is depleted – is a hallmark of a surface-coupled (LH) mechanism where nitrate reduction and H adsorption compete for sites.

Table S1. Specific surface area and pore volume analysis for CNF, CNF/TiO_2, and CNF/CNT supports obtained from the N_2 adsorption-desorption isotherms.

CNF Support	Specific Surface Area ($m^2\,g^{-1}$)				V_{tot} ($cm^3\,g^{-1}$)	Pore Volume Distribution (%)		
	S_{BET}	S_{EXT}	S_{micro}	S_{meso}		Micropore	Mesopore	Macropore
CNF	11 ± 1	9 ± 1	2 ± 0.1	6 ± 0.1	0.02 ± 0.005	5 ± 1	86 ± 5	9 ± 5
CNF/TiO_2	54 ± 1	47 ± 2	7 ± 3	50 ± 4	0.10 ± 0.001	3 ± 2	94 ± 3	3 ± 1
CNF/CNT	47 ± 3	41 ± 2	7 ± 2	31 ± 2	0.16 ± 0.006	2 ± 0.4	92 ± 0.2	5 ± 0.6

S_{BET}, Multi-point BET
S_{EXT}, External surface area (t-area) via the V-t method
S_{micro}, Microporous surface area where $S_{micro} = S_{BET} - S_{EXT}$
S_{meso}, Mesoporous surface area via the BJH method
V_{tot}, Total pore volume estimated at $P/P_0 \approx 0.95$

Table S2. Cu loading on the CNF supports as quantified by ICP-MS in terms of: Cu mass (mg_{Cu}), Cu mass with respect to electrode geometric area (A_{CNF} = 3 cm^2; mgCu cm^{-2}), and Cu mass with respect to electrode mass ($mg_{Cu}\,mg_{CNF}^{-1}$).

CNF Support	Catalyst Loading		
	mg_{Cu}	$mg_{Cu}\,cm^{-2}$	$mg_{Cu}\,mg_{CNF}^{-1}$
CNF	0.11 ± 0.02	0.04 ± 0.005	0.006 ± 0.002
CNF/TiO_2	0.09 ± 0.02	0.03 ± 0.008	0.018 ± 0.004
CNF/CNT	0.10 ± 0.02	0.03 ± 0.007	0.006 ± 0.005

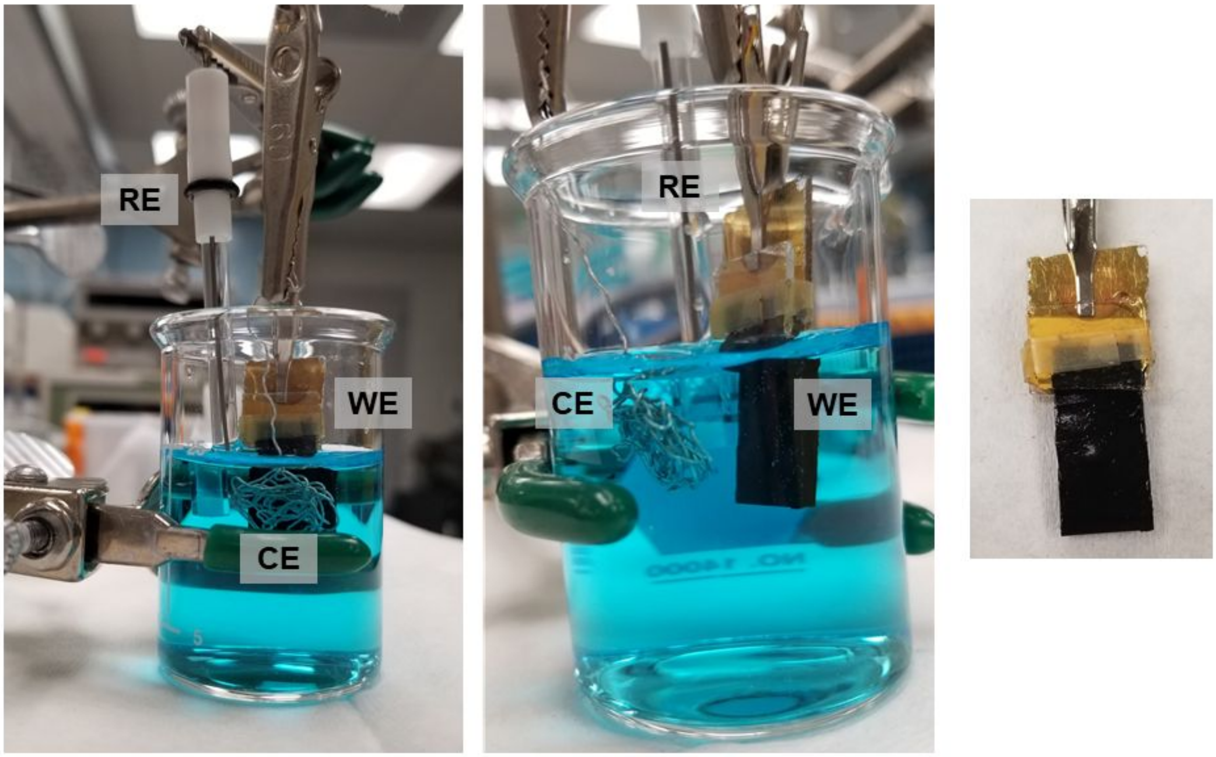

Figure S1. Photographs of the three-electrode system (with electrodes labeled) for the electrochemical deposition of Cu on a CNF sample (left, middle) and a CNF sample prepared for deposition (right).

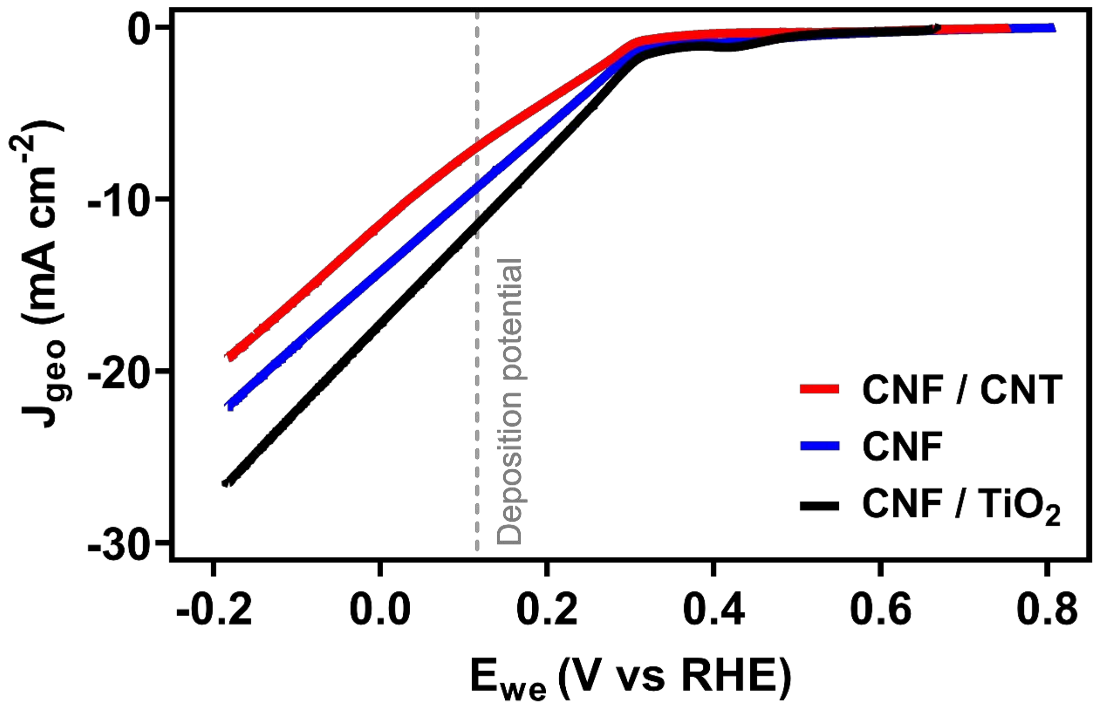

Figure S2. Current density (J_{geo}) versus applied potential (E_{we}) obtained via linear sweep voltammetry (LSV; 2 mV s^{-1}) with the three CNF substrates. LSV was conducted to determine the parameters for electrochemical deposition of Cu on the substrates. Deposition solution: 0.3 M Cu_2SO_4 + 0.1 M H_3BO_3, pH 2.0; RE: Ag/AgCl; CE: Pt wire.

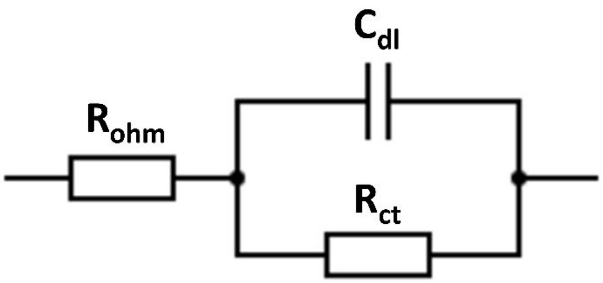

Figure S3. Representation of the Simple Randles model circuit, where R_{tot} is the total resistance, R_{ct} is the charge transfer resistance, and C_{dl} is the double-layer capacitance.

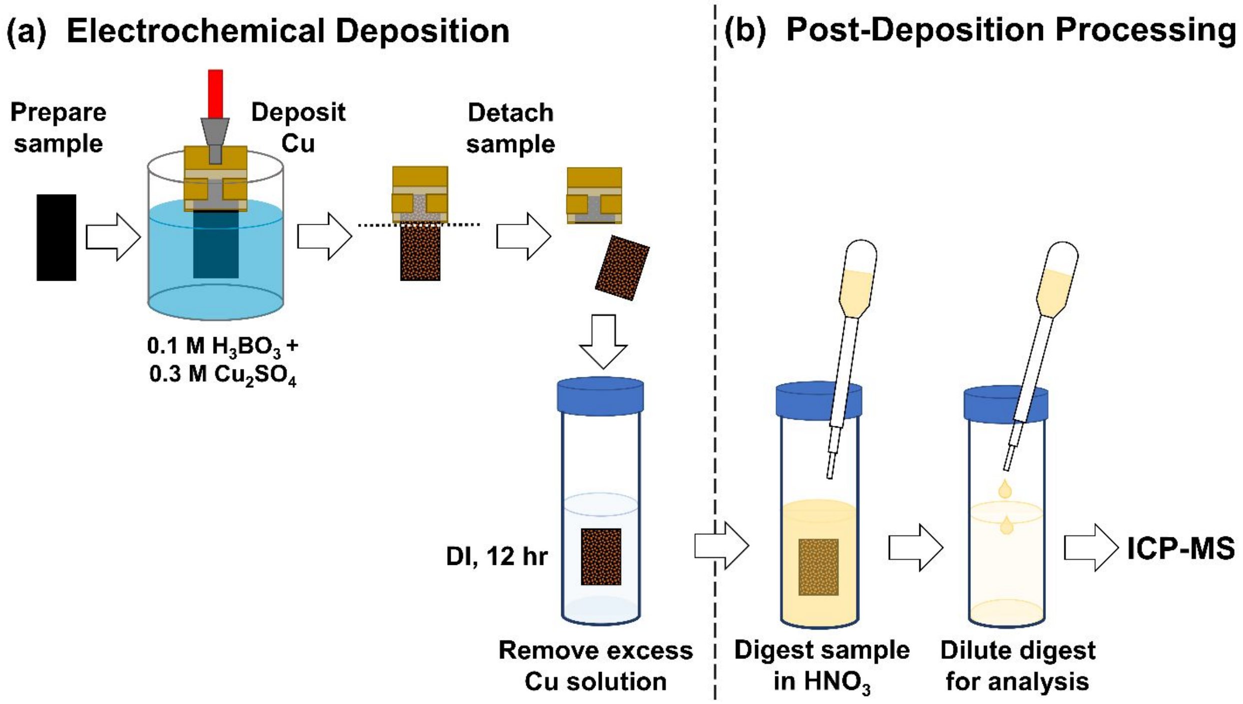

Figure S4. Schematic to illustrate how CNF electrodes were prepared for: (a) electrochemical deposition of copper and (b) ICP-MS to quantify copper mass loading following deposition.

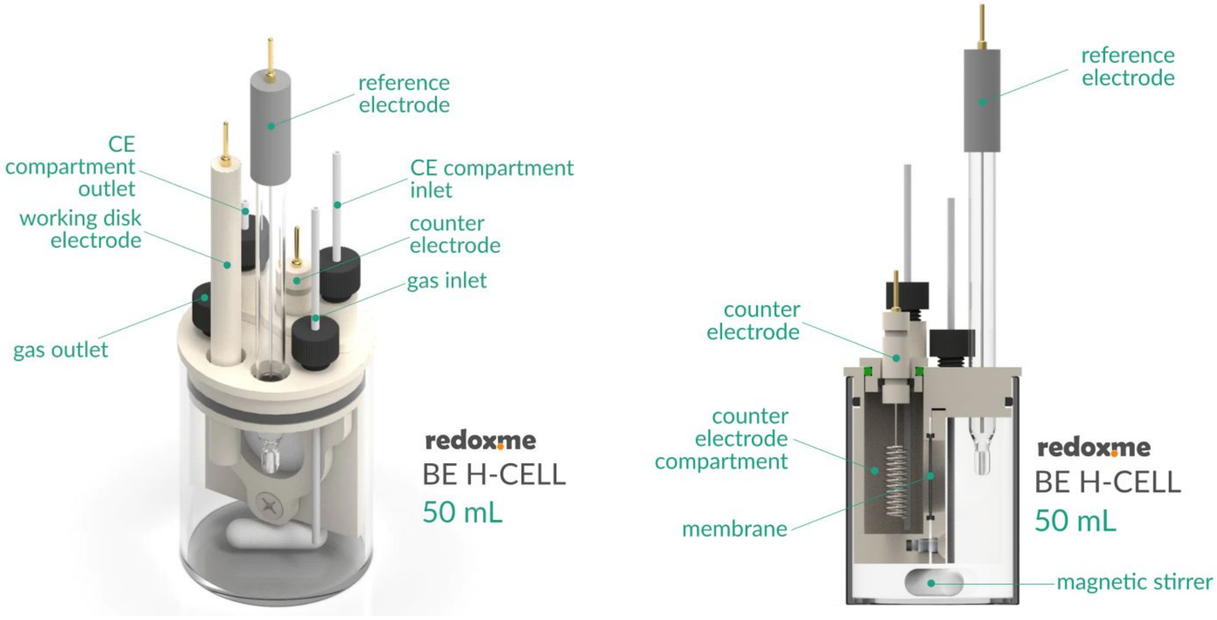

Figure S5. Digital renderings of the H-Cell reactor used for NO3RR experiments as provided by the vendor (figure provided by redox.me).

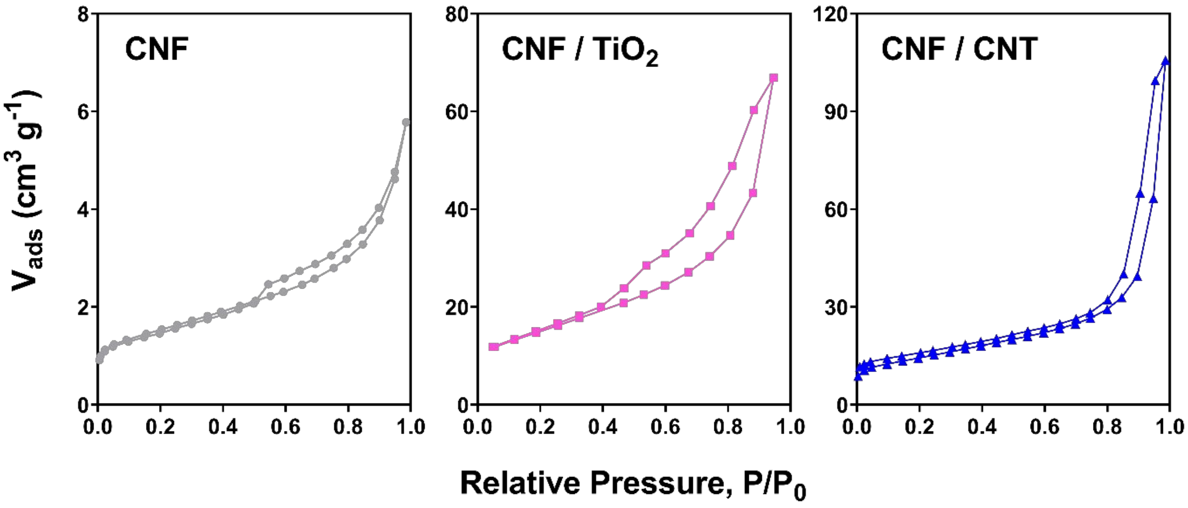

Figure S6. Brunauer–Emmett–Teller (BET) N_2 adsorption isotherms for CNF, CNF/TiO_2, and CNF/CNT, where the volume N_2 adsorbed = V_{ads} and relative pressure = P/P_0.

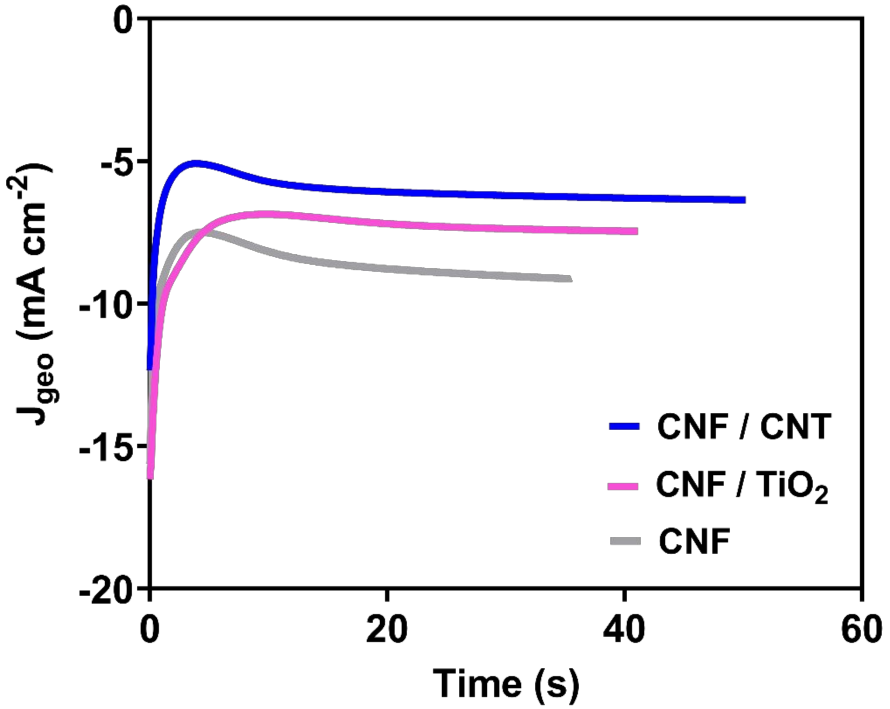

Figure S7. Representative current density-time (J_{geo} versus t) profiles for the electrochemical deposition of Cu on the CNF supports. Electrolyte: 0.3 M Cu2SO4 + 0.1 M H3BO3, pH 7.0; applied potential: -0.2 V vs Ag/AgCl; stopping criteria: 0.458 C; electrode area: 3.0 cm^2.

Table S3. Nitrate (NO_3^-) transformed (%) normalized to passed charge (mol NO_3^- consumed : mol e^- passed) for CNF/TiO_2/Cu, CNF/Cu, and CNF/CNT/Cu at the incremental charges 5, 10, 20, and 30 C. Experimental parameters: 500 ppm NO_3^- in 0.1M Na_2SO_4, pH 7.0, purged with Ar; stirred. Cu deposition at -0.2 V vs SCE, 0.458 C.

CNF Support	Applied Potential (V vs RHE)	NO_3^- Transformed (%)			
		5 C	10 C	20 C	30 C
CNF/TiO_2/Cu	-0.39	59 ± 8	53 ± 5	46 ± 3	N/A
CNF/TiO_2/Cu	-0.69	34 ± 11	29 ± 7	28 ± 5	24 ± 3
CNF/TiO_2/Cu	-0.79	28 ± 3	27 ± 1	23 ± 1	22 ± 2
CNF/Cu	-0.69	13 ± 3	19 ± 0	23 ± 5	22 ± 4
CNF/CNT/Cu	-0.69	2 ± 1	5 ± 1	5 ± 1	6 ± 1

Table S4. Faradaic efficiency (FE %) for copper-modified CNF supports with passed charge (5, 10, 20, 30 C). Experimental parameters: 500 ppm NO_3^- in 0.1M Na_2SO_4, pH 7.0, purged with Ar; stirred. Cu deposition at -0.2 V vs SCE, 0.458 C. Zero values indicate non-detect (no formation); N/A, not applicable. Only a single value was collected and validated for H_2.

CNF Support	Applied Potential (V vs RHE)	Product	Faradaic efficiency (%)			
			5 C	10 C	20 C	30 C
CNF/Cu	-0.69	NO_2^-	7.0 ± 6.5	23.1 ± 1.2	30.7 ± 13.8	27.3 ± 11.1
		NH_4^+	0	0	8.8 ± 12.4	22.2 ± 26.8
		N_2	92.8 ± 6.5	76.4 ± 1.2	59.5 ± 1.4	49.5 ± 16.4
		H_2	0.2	0.5	1.1	1.5
CNF/TiO_2/Cu	-0.39	NO_2^-	3.9 ± 15.2	5.4 ± 10.5	10.6 ± 10.2	N/A
		NH_4^+	0	0	0	N/A
		N_2	0	0	3.5 ± 10.2	N/A
		H_2	0	0	0	N/A
CNF/TiO_2/Cu	-0.69	NO_2^-	59.1 ± 27.4	44.8 ± 18.3	38.4 ± 12.7	27.9 ± 9.6
		NH_4^+	0	0	20.3 ± 14.5	42.4 ± 27.1
		N_2	6.7 ± 4.6	17.6 ± 6.1	18.0 ± 6.0	24.0 ± 17.5
		H_2	0.1	0.8	2.8	5.6
CNF/TiO_2/Cu	-0.79	NO_2^-	33.0 ± 0.4	29.3 ± 9.5	23.4 ± 5.2	20.6 ± 4.5
		NH_4^+	0	46.2 ± 65.3	59.9 ± 1.9	60.3 ± 7.1
		N_2	65.4 ± 8.2	35.2 ± 49.8	13.7 ± 1.5	15.3 ± 2.6
		H_2	0.1	0.1	0.6	1.1
CNF/CNT/Cu	-0.69	NO_2^-	4.5 ± 2.1	9.3 ± 2.6	10.7 ± 1.7	10.6 ± 0.6
		NH_4^+	0	0	0	4.0 ± 5.7
		N_2	N/A	N/A	N/A	N/A
		H_2	N/A	N/A	N/A	N/A

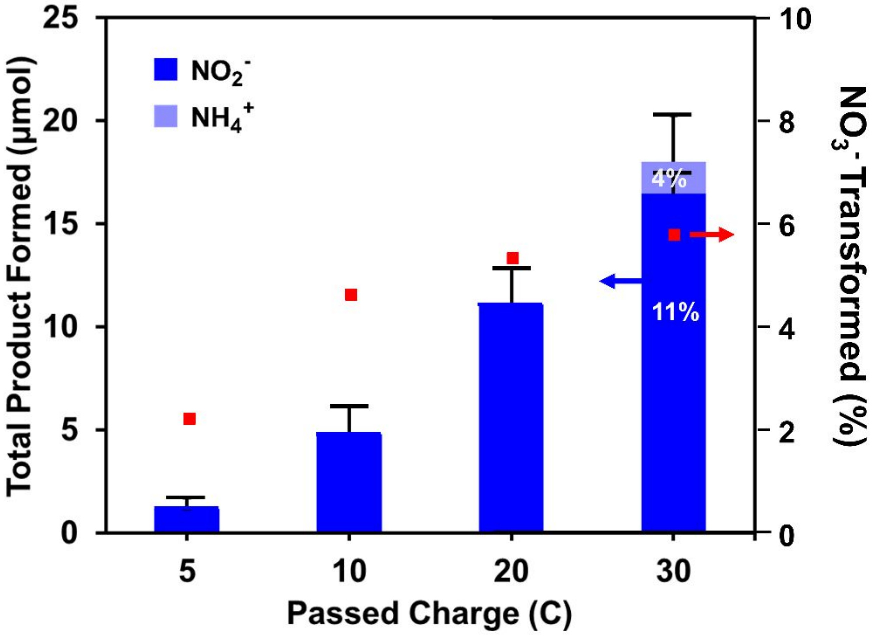

Figure S8. Ionic product distribution (primary axis) from nitrate (NO_3^-) reduction for CNF/CNT/Cu. NO_3^- transformed (%), normalized to passed charge, is provided on the secondary axis. Faradaic efficiency (FE %) for nitrite (NO_2^-) and ammonium (NH_4^+) at 30 C are displayed within the corresponding bar. Cu deposition at -0.2 V vs SCE, 0.458 C. Experimental parameters: 500 ppm NO_3^- in 0.1M Na_2SO_4, pH 7.0, purged with Ar; stirred.

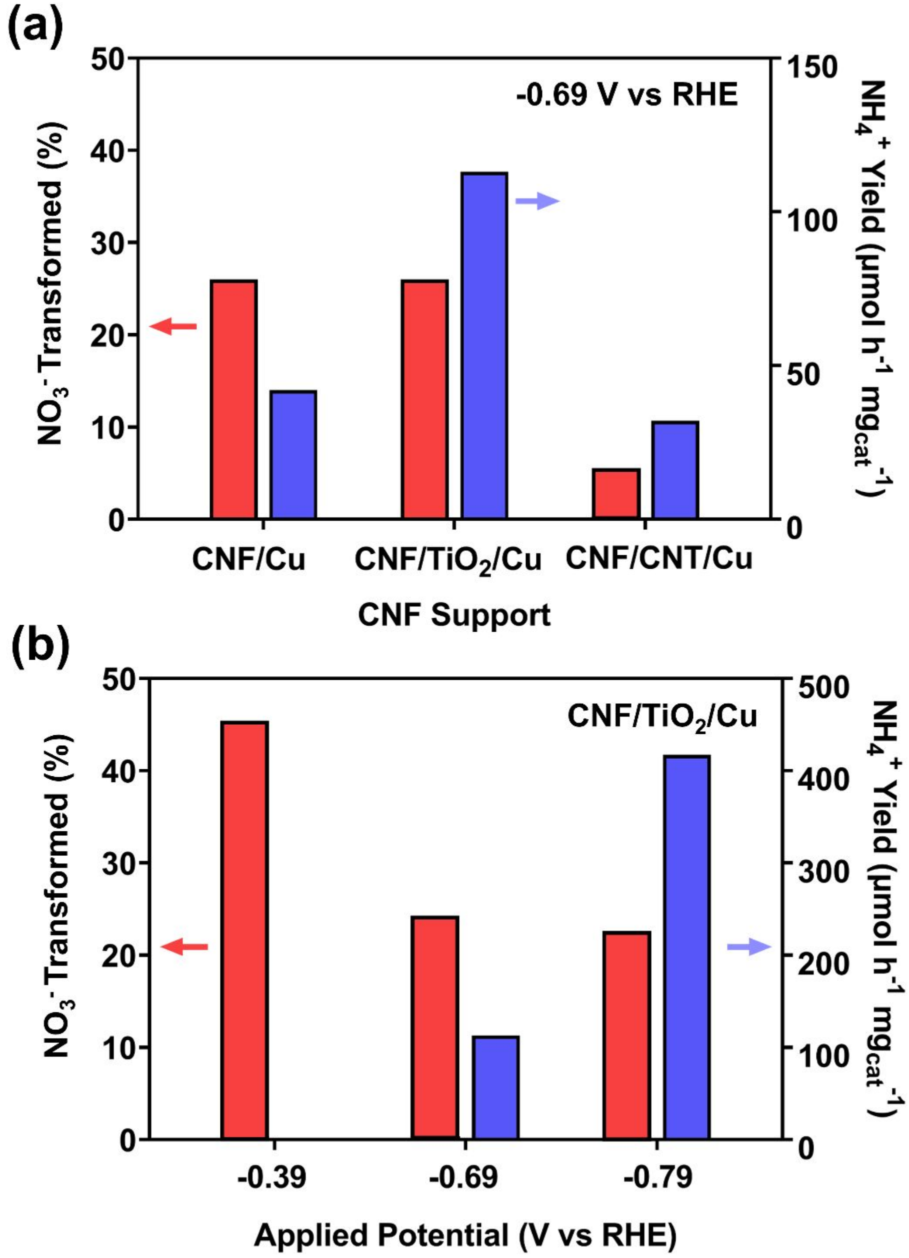

Figure S9. (a) Average NH_4^+ yield at -0.69 V vs RHE for the three CNF supports deposited with Cu. CNF/TiO_2/Cu excels at NH_4^+ production relative to CNF/Cu and CNF/CNT/Cu, producing upwards of 115 μmol h^{-1} mg_{cat}^{-1}. Although CNF/CNT/Cu produces relatively low NH_4^+ mass (2 μmol), CNF/CNT/Cu generates extremely high currents in a short amount of time to approach the NH_4^+ yield for CNF/Cu. (b) Average NH_4^+ yield for CNF/TiO_2/Cu at different applied potentials (-0.39, -0.69, -0.79 V vs RHE). The NH_4^+ yield agrees with the trend in NH_4^+ mass produced, where more negative potentials produced the greatest NH_4^+ mass and the greatest yield. From -0.69 to -0.79 V, the NH_4^+ yield for CNF/TiO_2/Cu increased over 3-fold. Cu deposition at -0.2 V vs SCE, 0.458 C. Experimental parameters: 500 ppm NO_3^- in 0.1M Na_2SO_4, pH 7.0, purged with Ar; stirred.

Table S5. Performance of other carbon-based electrodes for NO3RR.

Electrode Type (Cu–C composite)	Conditions / Comments	Potential (V vs RHE)	NH3 Faradaic Efficiency (FE, %)	NH3 Activity / Yield	Reference
Cu/CF	Graphite felt electrodeposited with Cu nanoparticles; scalable porous structure	–0.4 to –0.8	75–85	25–45 mA cm^{-2}	8
Cu–C aerogel	Carbon aerogel scaffold with atomically dispersed Cu sites	-0.9	~80	300 μg_{NH3} h^{-1} cm^{-2}	9
Cu@CNFs	Porous carbon nanofibers loaded with Cu nanoparticles	–1.2	~86	24.22 mg h^{-1} mg_{cat}^{-1}	10
Cu-Ppy	0.5 M H2SO4	-0.4	96	0.55 mmol h^{-1} cm^{-2}	11
Au–Cu NWs/CF	Nanowire Cu with Au doping on carbon felt	–1.05	84	5336 μg h^{-1} cm^{-2}	12
R-Cu2O/Cu/CF	N2-saturated 1M KOH+250mg L-1 NO3-	-0.25	84	2.17 mg cm^{-2} h^{-1}	13
Cu@C-800	0.1M Na2SO4+500 ppm NO3-	-0.9	78	51.7 ± 0.6 mmol h^{-1} g^{-1}	14
Cu2O/CuO @C	1.0M KOH+50 ppm NO3- (14.3 mg KNO3)	-0.25	93	0.045 mmol h^{-1} cm^{-2}	15
CuO/NF	50 mM Na2SO4+50 ppm NO3-+0.1 M NaCl	Constant current process	-	-	16
Cu2O/Cu NRs/CF	0.1 mol/L Na2SO4+30 mg/L KNO3	-1.4 V vs. Ag/AgCl	84	-	17
Cu (O)@CF-A	0.1M KOH+0.01M KNO3	-0.3	99.5	5.9 mg h^{-1} cm^{-1}	18
Cu/CuOx/CF	0.5M K2SO4+200mg L-1 NO3-	-1.3 V vs. SCE	94	0.22 mmol h^{-1} cm^{-2}	19
Cu2O h-NCs	0.5 M Na2SO4+50 mmol NaNO3	-0.85	93	56.2 mg h^{-1} $mgcat^{-1}$	20
Various carbon-based supports	See references within	–0.1 to -1.1	65-100 (in several studies)	Varied	21

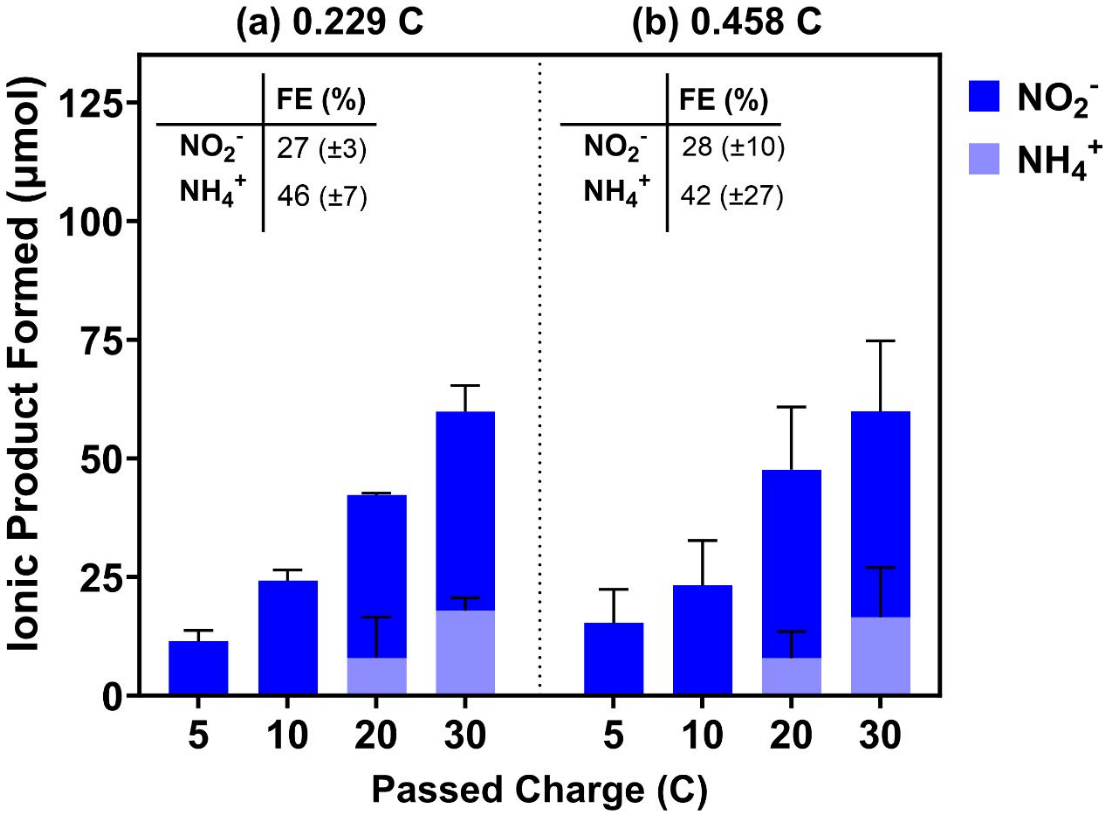

Figure S10. Ionic product distribution from nitrate (NO_3^-) reduction for CNF/TiO_2/Cu across Cu loading (a) 0.229 C and (b) 0.458 C. Average Faradaic efficiencies (FE, %) for nitrite (NO_2^-) and ammonium (NH_4^+) at 30 C are displayed in the insert. Cu deposition at -0.2 V vs SCE, 0.458 C. Experimental parameters: 500 ppm NO_3^- in 0.1M Na_2SO_4, pH 7.0, purged with Ar; stirred.

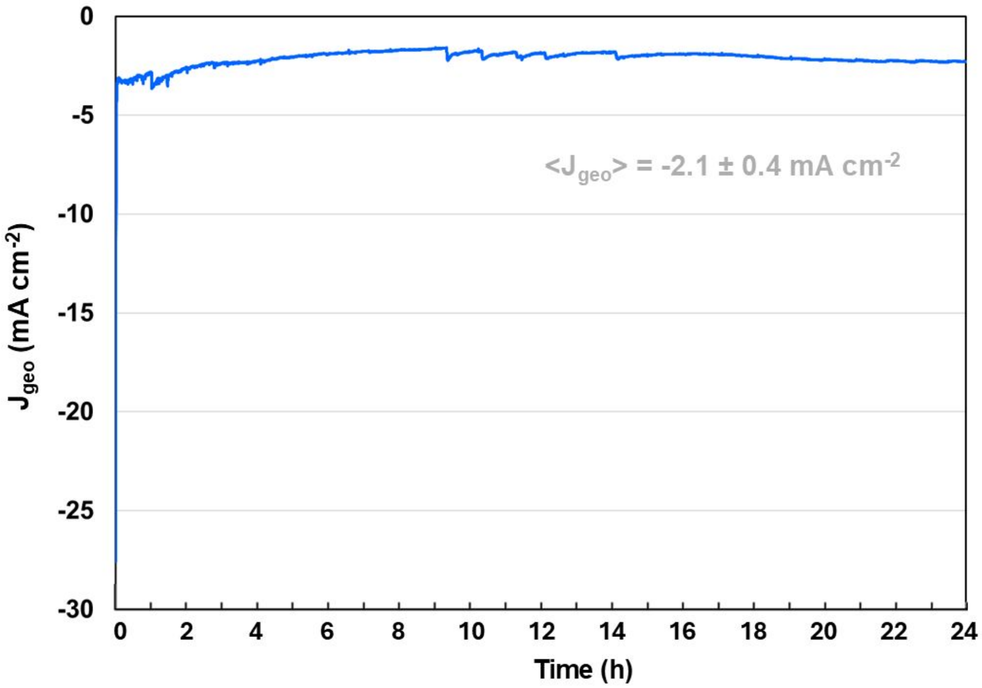

Figure S11. Current density-time (J_{geo} vs t) profile from nitrate (NO_3^-) reduction for CNF/TiO_2/Cu at -0.69 V vs RHE after 24 hours of continuous operation. The near-constant current density (< 10 % deviation) demonstrates excellent long-term electrode–catalyst stability. Cu depositon was carried at 0.12 V vs RHE, 0.458 C. Experimental parameters: 500 ppm NO_3^- in 0.1M Na_2SO_4, pH 7.0, purged with Ar; stirred.

References

1. AEROXIDE®, AERODISP®, and AEROPERL® Titanium Dioxide as Photocatalyst: Technical Information 1243 http://www.aerosil.com/sites/lists/RE/DocumentsSI/TI-1243-Titanium-Dioxide-as-Photocatalyst-EN.pdf (accessed January 29).

2. Peter, K. T.; Vargo, J. D.; Rupasinghe, T. P.; De Jesus, A.; Tivanski, A. V.; Sander, E. A.; Myung, N. V.; Cwiertny, D. M., Synthesis, Optimization, and Performance Demonstration of Electrospun Carbon Nanofiber-Carbon Nanotube Composite Sorbents for Point-of-Use Water Treatment. *ACS Appl Mater Interfaces* **2016,** *8* (18), 11431-40.

3. Peng, Y.; Zhang, F.; Xu, C.; Xiao, Q.; Zhong, Y.; Zhu, W., Adsorption of Nitrous Oxide on Activated Carbons. *Journal of Chemical & Engineering Data* **2009,** *54* (11), 3079-3081.

4. Zeng, Y.; Priest, C.; Wang, G.; Wu, G., Restoring the Nitrogen Cycle by Electrochemical Reduction of Nitrate: Progress and Prospects. *Small Methods* **2020,** *4* (12), 2000672.

5. de Groot, M. T.; Koper, M. T. M., The influence of nitrate concentration and acidity on the electrocatalytic reduction of nitrate on platinum. *Journal of Electroanalytical Chemistry* **2004,** *562* (1), 81-94.

6. Butzlaff, A. H.; Jensen, M.; Yan, C.; Ghanim, A.; Werth, C.; Cwiertny, D.; Mubeen, S., Electrospun TiO2/carbon composite nanofibers as effective (photo)electrodes for removal and transformation of recalcitrant water contaminants. *Environmental Science: Advances* **2023,** *2* (7), 967-981.

7. Kruk, M.; Jaroniec, M., Gas Adsorption Characterization of Ordered Organic−Inorganic Nanocomposite Materials. *Chemistry of Materials* **2001,** *13* (10), 3169-3183.
